# Establishment of a Conditionally Immortalized Wilms Tumor Cell Line with a Homozygous *WT1* Deletion within a Heterozygous 11p13 Deletion and UPD Limited to 11p15

**DOI:** 10.1371/journal.pone.0155561

**Published:** 2016-05-23

**Authors:** Artur Brandt, Katharina Löhers, Manfred Beier, Barbara Leube, Carmen de Torres, Jaume Mora, Parineeta Arora, Parmjit S. Jat, Brigitte Royer-Pokora

**Affiliations:** 1 Institute of Human Genetics and Anthropology, Heinrich-Heine University, D-40225, Düsseldorf, Germany; 2 Department of Oncology, Hospital Sant Joan de Deu, 08950 Barcelona, Spain; 3 Department of Neurodegenerative Diseases/ MRC Prion Unit, UCL, Institute of Neurology, Queen Square, London WC1N 3BG, United Kingdom; University of Bristol, UNITED KINGDOM

## Abstract

We describe a stromal predominant Wilms tumor with focal anaplasia and a complex, tumor specific chromosome 11 aberration: a homozygous deletion of the entire *WT1* gene within a heterozygous 11p13 deletion and an additional region of uniparental disomy (UPD) limited to 11p15.5-p15.2 including the *IGF2* gene. The tumor carried a heterozygous p.T41A mutation in *CTNNB1*. Cells established from the tumor carried the same chromosome 11 aberration, but a different, homozygous p.S45Δ *CTNNB1* mutation. Uniparental disomy (UPD) 3p21.3pter lead to the homozygous *CTNNB1* mutation. The tumor cell line was immortalized using the catalytic subunit of human telomerase (h*TERT*) in conjunction with a novel thermolabile mutant (U19dl89-97tsA58) of SV40 large T antigen (LT). This cell line is cytogenetically stable and can be grown indefinitely representing a valuable tool to study the effect of a complete lack of *WT1* in tumor cells. The origin/fate of Wilms tumors with *WT1* mutations is currently poorly defined. Here we studied the expression of several genes expressed in early kidney development, e.g. *FOXD1*, *PAX3*, *SIX1*, *OSR1*, *OSR2* and *MEIS1* and show that these are expressed at similar levels in the parental and the immortalized Wilms10 cells. In addition the limited potential for muscle/ osteogenic/ adipogenic differentiation similar to all other *WT1* mutant cell lines is also observed in the Wilms10 tumor cell line and this is retained in the immortalized cells. In summary these Wilms10 cells are a valuable model system for functional studies of *WT1* mutant cells.

## Introduction

Wilms tumor (WT), a malignant childhood neoplasm of the kidney, is thought to arise from embryonic renal mesenchyme with impaired nephrogenic differentiation potential. Most tumors have a mixed histology, containing blastema, epithelia and stroma. In the WT variant with a predominating stromal component, heterotypic cells, such as rhabdomyoblasts, fat, cartilage and bone can be found, not normally present in the kidney and likely to be derived from abnormal mesenchymal differentiation. Constitutional or somatic mutations in the *WT1* gene are found in most stromal-type tumors, often associated with mutations in the *CTNNB1* gene [1–5].

Intralobar nephrogenic rests (ILNR) occurring early in kidney development can be found as precursor lesions in *WT1* mutant tumors [6]. Microdissection of ILNRs in *WT1* mutant Wilms tumors revealed that these carry biallelic *WT1* mutations but no *CTNNB1* mutations, whereas the associated tumor cells had *CTNNB1* mutations [7]. Most *WT1* mutant tumors have additional mutations in *CTNNB1* or *WTX*, highlighting the importance of activated WNT signaling in tumors with inactive *WT1* [2,5,8]. The presence of activating mutations in *CTNNB1* or *WTX* suggests that the functional loss of *WT1* poses a strong selection pressure for additional mutations. This is further supported by our previous description of a patient with a germ line *WT1* mutation who developed four tumors with different *CTNNB1* mutations, suggesting their independent origin and/or tumor heterogeneity. In addition the same tumor harbored different *CTNNB1* mutations in different histological areas [9] (unpublished observation). In these Wilms tumors three “hits” occurred; the first is a germ line *WT1* mutation, the second is the loss of heterozygosity (LOH) in 11p, resulting in loss of the *WT1* wild type allele and the third is a *CTNNB1* mutation [9].

Most cell lines that we have established from *WT1* mutant Wilms tumors have additional *CTNNB1* mutations and the *WT1* mutation is either homozygous due to a mitotic recombination event or the cells have a deletion on one allele and a mutation in the other allele. The *WT1* gene is still present in all cell lines and theoretically a mutant RNA encoding a mutant protein can be synthesized. Indeed, we have recently shown that mutant WT1^Wilms3^ protein with a C-terminal extension (p.V432fsX87) exhibits gain of function properties. The mutant protein has lost the wild type WT1 function for sequence specific DNA binding, but facilitates the expression of genes regulating the cell cycle [10]. Therefore this mutant WT1 protein has not only lost its wild type function but has also gained a new function. It is of interest that Wilms3 cells do not carry a mutant *CTNNB1* gene; the gain of function of the mutant WT1 protein in regulating the cell cycle could explain, why these cells do not need additional mutations in *CTNNB1* nor *WTX* [10].

All our previously established *WT1* mutant Wilms tumor cell lines have a limited life span, similar to normal human mesenchymal stem cells (hMSC). Under in vitro growth conditions they can be cultivated at most for 60 population doublings. Primary cells in culture as well as some tumor cells have a limited life span that limits their use for experimental manipulation. Most biochemical and genetic studies require large cell numbers. Therefore, immortalized cell lines would be very useful for such studies. It has been described that the life span of normal human cells can be extended by introduction of the catalytic subunit of telomerase (TERT) [11]. More recent reports suggested that for a successful immortalization of human cells additional factors are needed. In many cases a temperature sensitive (ts) SV40 large T antigen (LT) was used resulting in conditionally immortalized cells. Successful immortalization using ts LT in combination with h*TERT* was described for many different normal cell types, for example glomerular mesangial cells, glomerular endothelial cells, podocyctes, mammary fibroblasts and endothelial cells, airway epithelial cells and hepatocytes [12–17]. All these normal cells did not proliferate at 37°C, the nonpermissive temperature of the tsLT, indicating that for their immortalization the LT is mandatory.

Here we describe a stromal type Wilms tumor with a homozygous deletion of *WT1* nested within a heterozygous 11p13 deletion and a *CTNNB1* mutation. Genetic analyses of cells derived from the Wilms tumor (Wilms10) showed the same *WT1*/11p13 alteration but a different mutation in *CTNNB1* as compared to the bulk tumor DNA. aSNP/CGH revealed UPD in 3p and 11p15 not extending to the *WT1* gene. Through immortalization of this cell line with ts LT and h*TERT*, we have established the first Wilms tumor cell line with a homozygous deletion of the entire *WT1* gene, resulting in a complete lack of *WT1*. This immortalized Wilms10 cell line should be useful for further exploration of the effect of *WT1* loss in genetic and biochemical studies and to further explore the origin and cell fate of Wilms tumors with *WT1* mutations.

## Methods

### Case description

A 2 year-old girl presented with an isolated left renal mass. The tumor was surgically removed upfront and the histology showed a triphasic stromal predominant Wilms tumor with focal anaplasia and p53 over-expression in these anaplastic foci (S1 Fig). The tumor infiltrated the renal capsule, the renal sinus, but did not invade the vessels or the ureter. Regional lymph nodes had no evidence of tumor. Procedures were approved by the Institutional Review Board (IRB) at Hospital Sant Joan de Déu (Barcelona, Spain) and are in accordance with the principles expressed in the Declaration of Helsinki. Written informed consent was obtained from parents. Tumor material necessary for histologic and molecular diagnosis was obtained from the tumor before a portion was processed for research purposes, following Standard Operating Protocols at the Department of Pathology.

### Mutation analysis

Tumor DNA was isolated directly from a piece of solid tumor tissue. DNA from blood and cell culture cells was isolated by standard methods. The complete *WT1* gene and *CTNNB1* exon3 was analyzed as described before [5].

P53 mutation analysis was done by Next-generation pyrosequencing using 454 Titanium Amplicon chemistry (Roche Applied Science, Penzberg, Germany) according to the manufacturer's instructions (8 amplicons, Transcript-ID ENST00000269305). Primer sequences and PCR conditions were kindly provided by A. Kohlmann (MLL, München). Sequencing run of the *TP53* gene was carried out on a GS Junior System instrument (Roche Applied Science). All sequencing data were generated using the Junior Sequencer Instrument software version 2.7 (Roche Applied Science). Sequence alignment and variant detection were performed using the GS Amplicon Variant Analyzer software version 2.7 (Roche Applied Science).

### Cell culture and establishment of immortalized cells

The tumor was finely minced and a cell culture was established using MSCG medium as described [18]. According to our nomenclature of cells established from stromal-predominant Wilms tumors, this cell culture was called Wilms10.

To establish an immortalized cell line, the catalytic subunit of human telomerase (h*TERT*, pBABE-hygro-h*TERT*, Addgene 1773) [19] and a novel triple mutant SV40 LT (U19dl89-97tsA58) was introduced into primary Wilms10 cells via retroviral infection as described [15]. The triple mutant is a combination of three SV40 early region mutations: U19, prevents T antigen from binding SV40 origin like DNA sequences [20]; dl89-97, a deletion of amino acids 89–97 that prevents binding to Bub1 [21]; and tsA58, that confers thermolability [22,23]. It was prepared by replacing an early region fragment encompassing the dl89-97 mutation and the large T antigen splice junction within pZipSVU19tsA58 early region to generate the novel thermolabile triple mutant LT antigen cDNA (PA and PSJ, unpublished)[15]. The pZipNeoU19dl89-97tsA58 was used to derive a stable amphotropic TEFLY-A producer cell line as described previously (PA and PSJ, unpublished)[15]. Amphotropic viruses prepared from the stable producer cells for tsLT and h*TERT* from a triple transfected HEK293FT cell line were mixed and used to transduce the primary Wilms 10 cells. After infection, the cells were cultured in G418 (selection for tsLT, 200 μg/ml) for 1 week, followed by hygromycin (selection for h*TERT*, 20 μg/ml) for another week to select for successfully doubly transduced cells. Single cell clones were isolated and cultivated at 33°C initially to maintain the active form of the temperature sensitive LT. After the first confluence, clones were split and cultured at 33° and 37°C. One immortalized clone was selected for further studies and was named imWilms10. The imWilms10 cells continued to grow at 37°C, whereas normal hMSC immortalized with the same genes did not continue to proliferate at 37°C. The imWilms10 cells were continuously cultured in medium with alternating hygromycin and G418 for one week each, with a break of selection for one week.

### SNP/CGH array analysis and custom array

The SNP/CGH array (aSNP/aCGH) analysis of the primary tumor and the tumor cell culture from patient Wilms10 was performed using a 2x400K oligonucleotide microarray (Sure Print G3 Human Genome CGH + SNP Microarray; Agilent Technologies Alto, CA, USA). With this array format, copy number changes, as well as copy neutral aberrations, such as uniparental disomy (UPD) can be detected on the same array. The samples were prepared and labeled as described by the manufacturer (Protocol Version 7.3 March 2014). To detect uniparental disomy (UPD), a female gender reference DNA (NA12878, Coriell Institute, USA) has to be used as control. To quantify the array data the feature extraction module of Agilent's CytoGenomics software (Version 1.5.1.0) was used and, for visualization, CytoGenomics Version 2.0.6.0.

To determine the breakpoints of the homozygous *WT1* and heterozygous 11p13 deletion, a high-resolution array was designed (http://earray.chem.agilent.com/earray/). The region of the homozygous *WT1* alteration was covered with oligonucleotides at a distance of 100 bp, whereas the probes for the heterozygous deletion had a spacing of 300 bp.

### Protein analysis

For the analysis of tsLT and MEST, proteins were extracted from the cells using RIPA buffer (150mM NaCl, 1% NP40, 0.5% DOC, 0.1% SDS, 50 mM Tris (pH8.0)), separated by SDS-PAGE and transferred to a PVDF membrane. The blot was incubated with a monoclonal antibody against SV40 LT (MAb423) or a monoclonal antibody against MEST (Abcam, 151564).

For the simultaneous analysis of the phosphorylation status of 49 tyrosine kinase receptors a ProteomeProfiler^TM^ antibody array was used. Proteins from Wilms10 and imWilms10 cells cultured at 33°C were extracted and incubated with the antibody arrays as described by the manufacturer (R&D systems).

### Differentiation analysis

Adipogenic and osteogenic differentiation was analyzed using reagents from Lonza and as described by the manufacturer. hMSC cells were used as controls in the same experiments. Non-induced control cells were kept in the respective maintenance media. After a 3 week induction and a maintenance phase, cells were processed for adipogenic differentiation by staining with Oil Red O. In addition cells were cultured for 10 days in the induction and maintenance medium for adipogenesis and total RNA (RNeasy, Qiagen) was extracted at day 0 and 10 for Q-RT-PCR analyses. Assay on Demand for *PPARG* (Hs01115513_m1, Life technologies) was used for the analysis of adipogenesis [24]. Immortalized cells were cultured in the differentiation medium for 18 days before RNA isolation.

For osteogenic differentiation cells were harvested after 19 days of differentiation by scraping them in the presence of 0.5M HCl and a calcium Liquicolor assay was performed according to the manufacturers instructions (Stanbio Laboratory, USA).

Myogenic differentiation was induced with DMEM/F12 (Gibco) supplemented with 2% horse serum for 12 days and HSMM cells were used as controls. For immunofluorescence analysis cells were seeded in four chamber slides (BD Biosciences) and after growth in induction or control medium for 9 days, cells were stained with a Titin-specific antibody as described before [18].

### Gene expression analysis

For gene expression analyses total RNA was isolated from the Wilms10 tumor cell line and the immortalized cells (imWilms10) cultured at 33°C using the RNeasy Mini Kit (Qiagen). RNA from two biological replicates was analyzed in all gene expression experiments. RNAs were labeled in the One-Color format as described by the manufacturer (Agilent Technologies) and hybridized to 4x44K”Whole Human Genome Oligo Microarrays (V1, Agilent)" in the presence of “Spike-In positive controls” (Agilent). The microarray scans were quantified using Agilent Feature Extraction Software (V10.1.1.1). Basic statistical analyses were performed within the 'R' statistical computing environment (R Development Core Team, 2011). The R-library 'Limma' [25] Bioconductor (www.bioconductor.org) was used for quantile normalization of microarrays [26]. To compare primary and immortalized cells, gene expression data were first filtered for genes showing a minimal intensity of 200 in at least one channel. Statistical significance of single genes was determined using Limma's moderated t-test, with a significance level of 0.1 applied to the FDR adjusted p-values. Additionally, only genes showing an absolute fold change of >1.5 were considered relevant. For further less stringent comparisons, e.g. pathway analyses not requiring all genes involved to show a significant differential expression we extended our gene selection to include genes with a raw p-value of up to 0.05, same expression and fold change cut-off as above. Gene expression data can be found at GEO: GSE 71265

For Q-RT-PCR analyses cDNAs were synthesized using TaqMan Reverse Transcription Reagents (Applied Biosystems). The Q-RT-PCR experiments were performed in triplicates using the TaqMan gene expression assay probes from Applied Biosystems for *WT1* (Hs00240913_m1) and *IGF2* (Hs01005963_m1) with Brilliant II QPCR Master Mix with Low Rox (Agilent Technologies). The expression levels were normalized with *RER1* (Hs00199824_m1) RNA. The reason for using *RER1* as calibrator was that this gene was basically never regulated in any of our gene expression studies. The Q-RT-PCRs experiments were run on a Mx3000P Sequence Detection System (Stratagene). Statistical significance of the normalized Ct values (ΔCt) was assessed by a t-test.

## Results

### *WT1* analysis and aSNP/CGH

**Fig 1:**
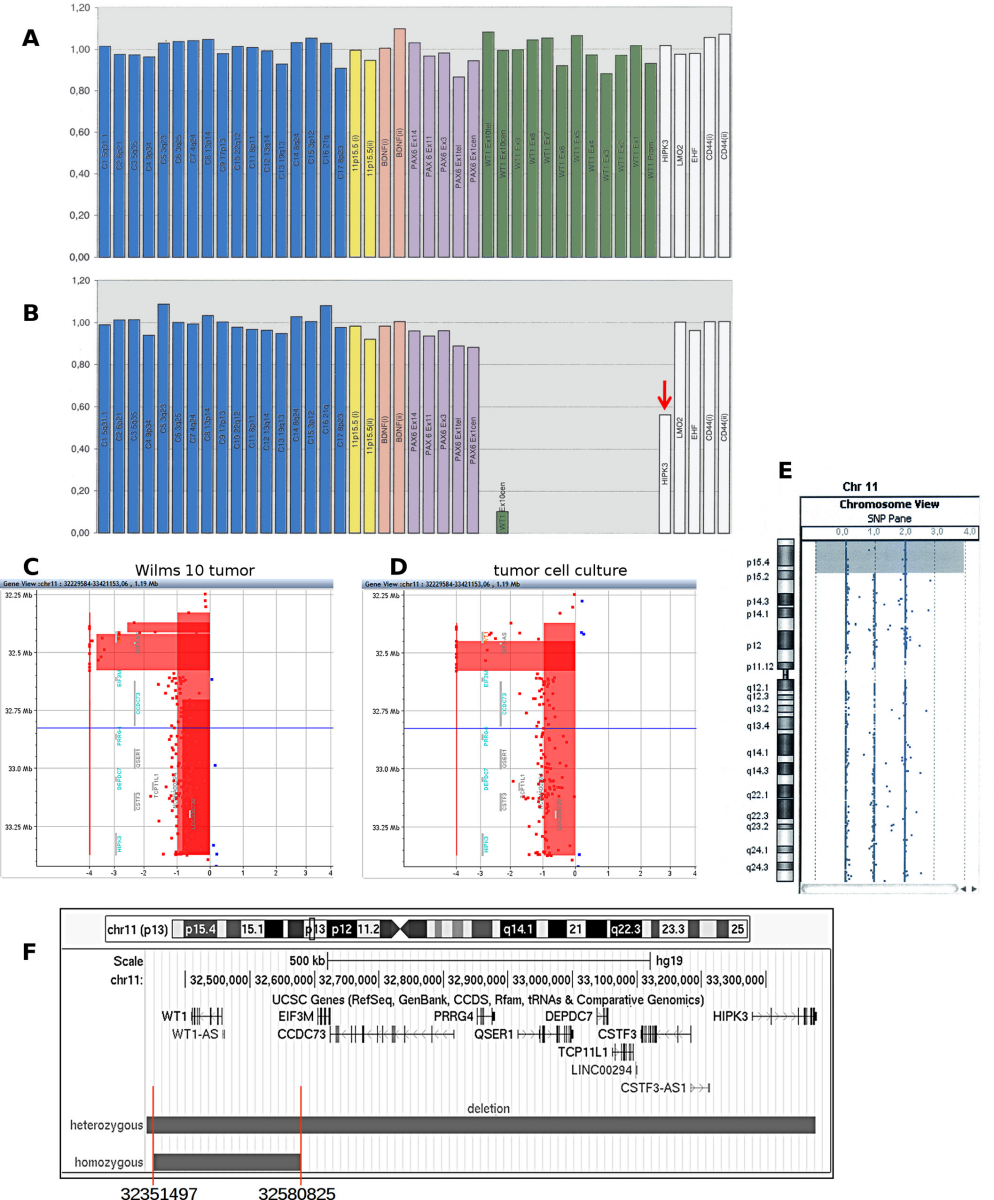
Characterization of the chromosome 11p13 alteration in tumor and tumor cell culture DNA from patient Wilms10. (A) Multiplex ligation-dependent probe amplification analysis (MLPA) of blood DNA. Green peaks are derived from *WT1* exons, white peaks represent genes proximal of *WT1* in 11p13, purple peaks correspond to *PAX6* and pale pink peaks to *BDNF* exons, distal of *WT1* in 11p13. The yellow peaks represent 11p15 markers and blue peaks are controls from different chromosomes. (B) In DNA isolated from the primary Wilms10 tumor; only one small peak from exon 10 of *WT1* is observed, whereas all other products from *WT1* are missing entirely. A red arrows indicates the reduced peak from the *HIPK3* gene. (C) aSNP/aCGH data of the 11p13 region from the primary Wilms tumor and (D) from the tumor derived cell culture. The homozygous deletion covering the *WT1* gene is clearly visible with log ratio of -4. (E) UPD limited to 11p15 as shown with the cytogenomics workbench program. (F) Summary of the genomic alterations in 11p13 with the positions of the heterozygous and homozygous deletions.

Using DHPLC and DNA sequencing, no pathogenic mutation of *WT1* was found in blood or tumor DNA. Multiple-ligation probe analysis (MLPA,) did not identify copy number variations in the blood DNA (Fig 1A), whereas the tumor DNA carried a homozygous deletion of the *WT1* gene (Fig 1B). This result suggests that the tumor DNA preparation contained some DNA from normal cells that resulted in a wild-type *WT1* DNA sequence. In contrast, by using MLPA we did not detect amplification of contaminating normal DNA, except for one small peak, corresponding to exon10 of *WT1*. The peak for a neighboring gene, *HIPK3* was reduced, suggesting a heterozygous deletion (Fig 1B, red arrow). This indicates that the patient had a tumor specific homozygous *WT1* deletion and a heterozygous deletion extending at least to the *HIPK3* gene.

To study the deletion in more detail a combined aSNP/aCGH analysis was performed with DNA isolated from the tumor, co-hybridized with reference control DNA to detect copy number imbalances. This analysis revealed that the homozygous deletion was restricted to the *WT1* gene and a few neighboring oligonucleotides with a size of 228kb (log ratio >4) (Fig 1C). The heterozygous deletion had a size of 1.05 Mb and encompasses the genes *EIF3M*, *CCDC73*, *PRRG4*, *QSER1*, *DEPDC7*, *TCP11L1*, *CSTF3 and HIPK3*. In addition, two deleted segments were identified on chromosome 1p32p32.1 with a maximal size of 3.2 Mb (57288535–60440170) and a smaller deletion at 1p31.1 with a maximal size of 203kb (74946777–75185022) (S2A and S2B Fig). Furthermore, two UPD regions could be identified with this array format; one limited to 11p15.3pter containing *IGF2*, but not extending to the *WT1* gene (219089–13058405, 12.8Mb) (Fig 1E and S3 Fig) and another one at 3p21.3pter (107994–49449638, 49.3Mb), the interval where the *CTNNB1* gene is located (S4 Fig).

DNA from the established tumor cell line (Wilms10) was also analyzed with aCGH/aSNP and the same 11p13 heterozygous/homozygous deletion was identified (Fig 1D). A cytogenetic analysis of the tumour cell culture showed a normal karyotype, 46, XX (not shown), indicating that the 1 Mb deletion in 11p13 and the 3.2 Mb deletion in 1p32.2p32.1 is below the cytogenetic detection limit.

A custom array covering the heterozygous/homozygous deleted 11p13 segment at a high density revealed the start and endpoint position of the heterozygous/homozygous deletions in the tumor and the established Wilms10 tumor cell line (S5 Fig). Fig 1F shows an overview of the exact deletion on chromosome 11p13. Taken together the tumor contained a complex and unusual abnormality of chromosome 11 with a region of UPD restricted to 11p15 and a homozygous deletion restricted to the *WT1* gene within a heterozygous 11p13 deletion. The derived cell culture harbored the same alterations as the tumor sample.

**Fig 2:**
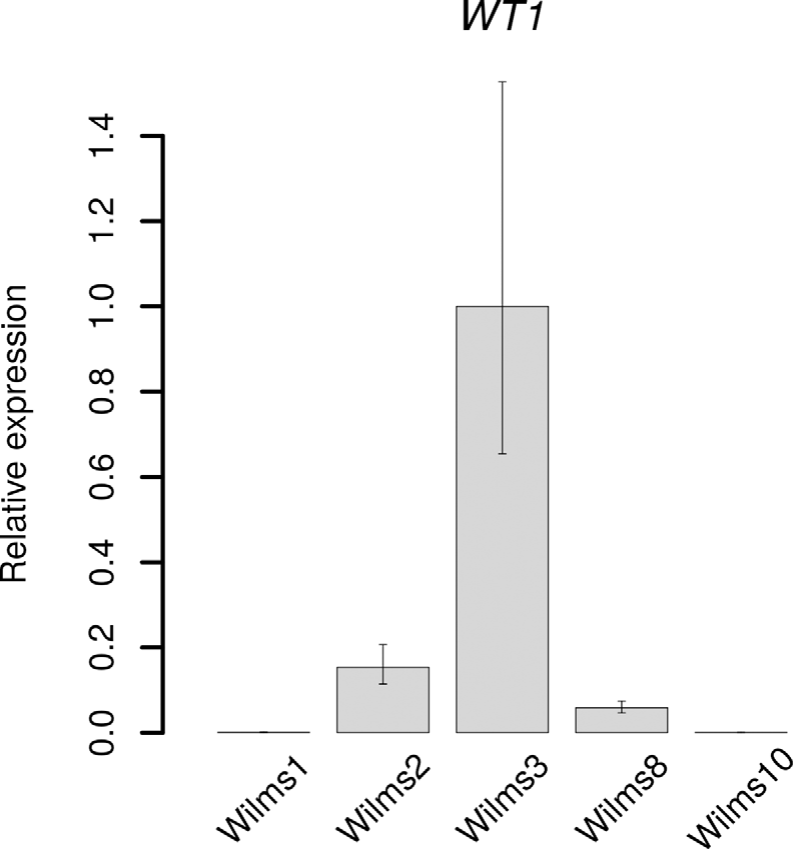
Quantitative RT-PCR analysis of the *WT1* expression in various WT cell lines. Total RNA from the *WT1* mutant cell lines Wilms1, Wilms2, Wilms3, Wilms8 and Wilms10 was analyzed by Q-RT-PCR. The genetic alterations present in these cell lines are found in S1 Table. The expression level was normalized versus *RER1*, a gene with the lowest variation between the cell lines in our gene expression studies. The analysis was performed in triplicates. The error bars correspond to 95% confidence intervals. The relative expression is shown versus Wilms3.

As this is the first cell line with no genomic DNA covering the *WT1* gene it was of interest to compare the level of *WT1* gene expression in the cell lines with the different *WT1* mutations versus the Wilms10 cell line. The genetic characteristics of all established cell lines is shown in S1 Table. As expected the Q-RT-PCR analysis showed that Wilms10 cells do not express any *WT1* mRNA and that the level varies between cell lines with the lowest, almost undetectable level in Wilms1 cells and the highest level in Wilms3 (Fig 2). It will be interesting to explore the difference in gene expression profiles between the different WT cell lines depending on the level of *WT1* in future studies but this is not within the scope of this work.

### *CTNNB1* and TP53 analysis

**Fig 3:**
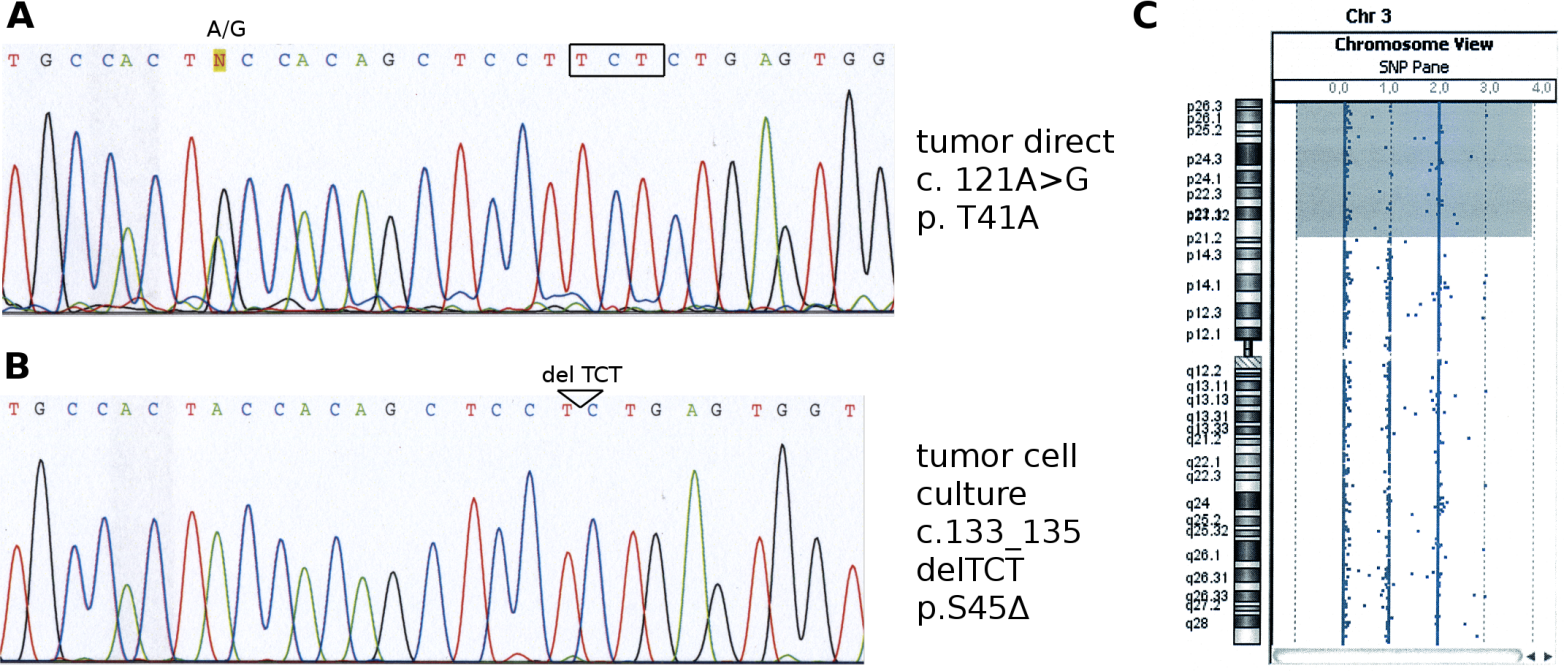
Comparison of *CTNNB1* mutations and UPD region on chromosome 3 in the primary Wilms10 tumor and tumor-derived cells. (A) DNA sequencing reveals a heterozygous A>G *CTNNB1* mutation at position c.121/p.T41A in the primary Wilms10 tumor. The position of the three deleted nucleoties in the tumor cells culture are boxed. (B) identification of a homozygous deletion c.133_135del TCT (p.S45Δ) in the *CTNNB1* gene in the Wilms10 tumor-derived cell line. The position of this deletion is indicated above the DNA sequence. (C) UPD region on chromosome 3p as shown with the cytogenomics workbench program.

The primary tumor harbored a heterozygous c.121A>G/p.T41A *CTNNB1* mutation (Fig 3A), whereas the Wilms10 tumor cell culture carried a homozygous deletion of three nucleotides c.133-135 del TCT, leading to loss of amino acid S45 (p.S45Δ) (Fig 3B). The p.T41A *CTNNB1* mutation identified in the tumor specimen was not seen in the cell culture. As cultured tumor cells have two copies of chromosome 3 (data not shown) and UPD of 3p (Fig 3C) this indicates that the homozygous p.S45Δ mutation in the cell culture is the result of a mitotic recombination with loss of the normal *CTNNB1* allele and duplication of the mutant allele. These two different mutations in the tumor specimen and the tumor cell line point to tumor heterogeneity. Furthermore as the tumor and the cell line have the same *WT1* alteration but different *CTNNB1* mutations this supports the model that the *WT1* alteration occurs first, with a high selection pressure to acquire mutations in *CTNNB1*.

No mutation was identified in TP53 in the tumor DNA nor in the tumor cell culture DNA. However, DNA from the anaplastic focus was not available and a mutation might be present in these cells (S1 Fig).

### Immortalization of Wilms10 and characterization of imWilms10 cells

All established Wilms tumor derived cell lines from tumors with *WT1* mutations as well as Wilms10 cells have a limited in vitro growth potential. Therefore we wished to immortalize Wilms10 cells with the homozygous *WT1* deletion using a tsLT in conjunction with human telomerase (hTERT) enabling their long term passaging. For this purpose an expression construct encoding a novel triple mutant of LT (U19dl89-97tsA58) was used; this mutant T antigen does not bind to ori-like DNA sequences, does not bind BUB1 and is thermolabile [20,21,23]. When this mutant tsLT was used in conjunction with an expression construct encoding h*TERT*, immortalized cells were readily established and these were termed "imWilms10". The cells were cultured so far for more than 30 passages, corresponding to 90 population doublings. Cytogenetic analyses of immortalized cells revealed a normal karyotype (not shown). The immortalized cell line remained cytogenetically normal for many passages, possibly due to the deletion of the amino acids responsible for BUB1 binding [21,27]. aCGH showed that the immortalized cells harbor the same heterozygous/homozygous 11p13 alteration with a complete loss of *WT1* and the two deletions on chromosome 1 (not shown).

To demonstrate that the tsLT protein is expressed in these cells, they were cultured at 33° (permissive temperature) and 39°C (nonpermissive temperature) and protein was extracted from these cells. Although the tsLT construct encoded a ts mutant protein a low level was detected at 39°C (S6A Fig). Sequencing of the tsLT insert revealed the correct presence of the ts mutation in the LT gene (PSJ, not shown). Similar observations were made in other immortalized WT cells using the same T antigen construct (unpublished Görldt, Tenbusch and BR-P). h*TERT* expression in these cells was verified by RT-PCR (S6B Fig).

**Fig 4:**
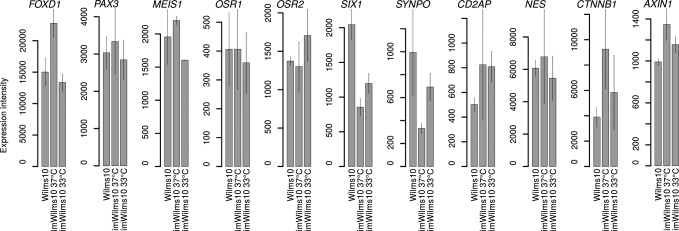
Similar expression level of selected kidney marker genes in imWilms10 and Wilms10 cells. The selection of these genes was based on studies of gene expression in various compartments during kidney development. All of these genes are also expressed in the other *WT1* mutant cell lines that we have previously established. The data are derived from microarray analyses of two biological replicates. The expression level is indicated from microarray intensity and the bar represents standard error.

In order to evaluate the effect of the immortalization process of Wilms10 cells by h*TERT* and tsLT we established gene expression profiles of cells cultivated at 33°C where both genes are active (permissive temperature for tsLT) using Agilent whole genome microarrays. It was the aim of this experiment to investigate the impact of the expression of these two genes on the transcriptome of Wilms10 tumor derived cells. This information shows whether the “biology” of immortalized (imWilms10 cells) is affected. However, it should be noted that 33°C is a nonphysiological temperature for human cells and they grow very slowly at that temperature. First we compared the expression levels of selected genes that are expressed in early kidney development and/or different compartments of the kidney [28]. These genes are also expressed in the other *WT1* mutant cell lines [18]. For example, *FOXD1* is typically expressed in stromal cells and also in all *WT1* mutant tumor derived cell lines we have established previously. Fig 4 shows that Wilms10 and imWilms10 cells express similar levels of *FOXD1* mRNA. Likewise *PAX3* is expressed in stromal cells and ectopically in WT with a myogenic histopathology [29]. High expression levels are found in our *WT1* mutant cell lines [18] and in Wilms10 and immortalized imWilms10 cells (Fig 4). The expression levels of genes involved in early kidney differentiation e.g. *MEIS1*, *OSR1*, *OSR2* and *SIX1* [28] are not affected by tsLT and h*TERT* expression (Fig 4). Likewise expression levels of kidney specific genes *SYNPO*, *CD2AP*, *NES* and genes associated with active Wnt signaling like *CTNNB1* and *AXIN1* are similar in Wilms10 and imWilms10 cells (Fig 4). We conclude that tsLT and h*TERT* dependent immortalization does not affect the expression of these characteristic kidney marker genes in Wilms10 cells.

**Fig 5:**
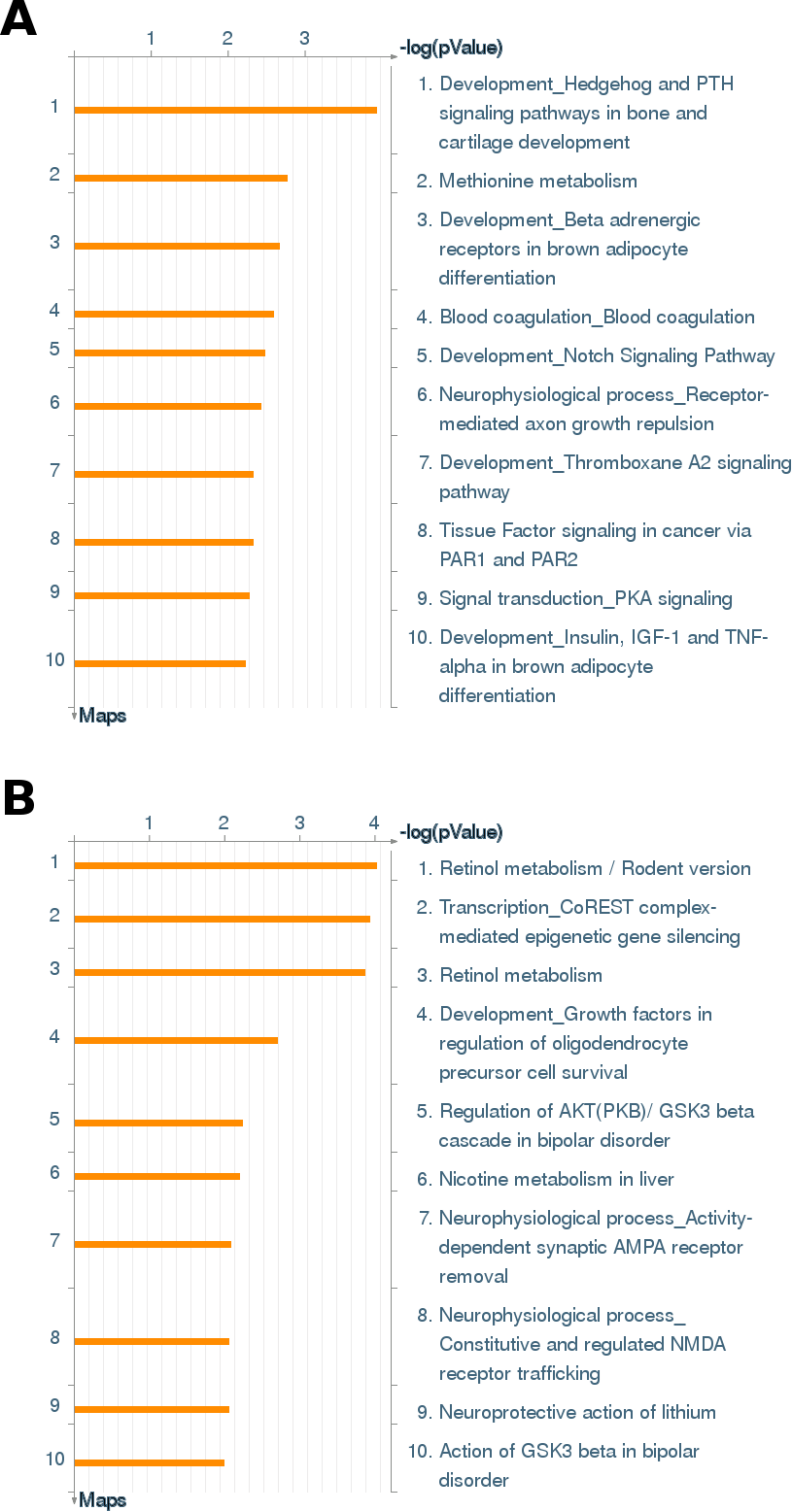
Comparison of gene expression of the imWilms10 cells cultured at 33° C with non immortalized Wilms10 cells. The data set of differentially expressed genes was analyzed with the MetaCore algorithm "pathway analysis" to identify significantly enriched pathways. In this analysis the differentially expressed gene set of up- and down-regulated genes were analyzed separately (FDR adjusted p-value of 0.1). (A) the top 10 enriched pathways for down-regulated genes. The log P-values are indicated above the list. (B) the top 10 enriched pathways for up-regulated genes.

**Fig 6:**
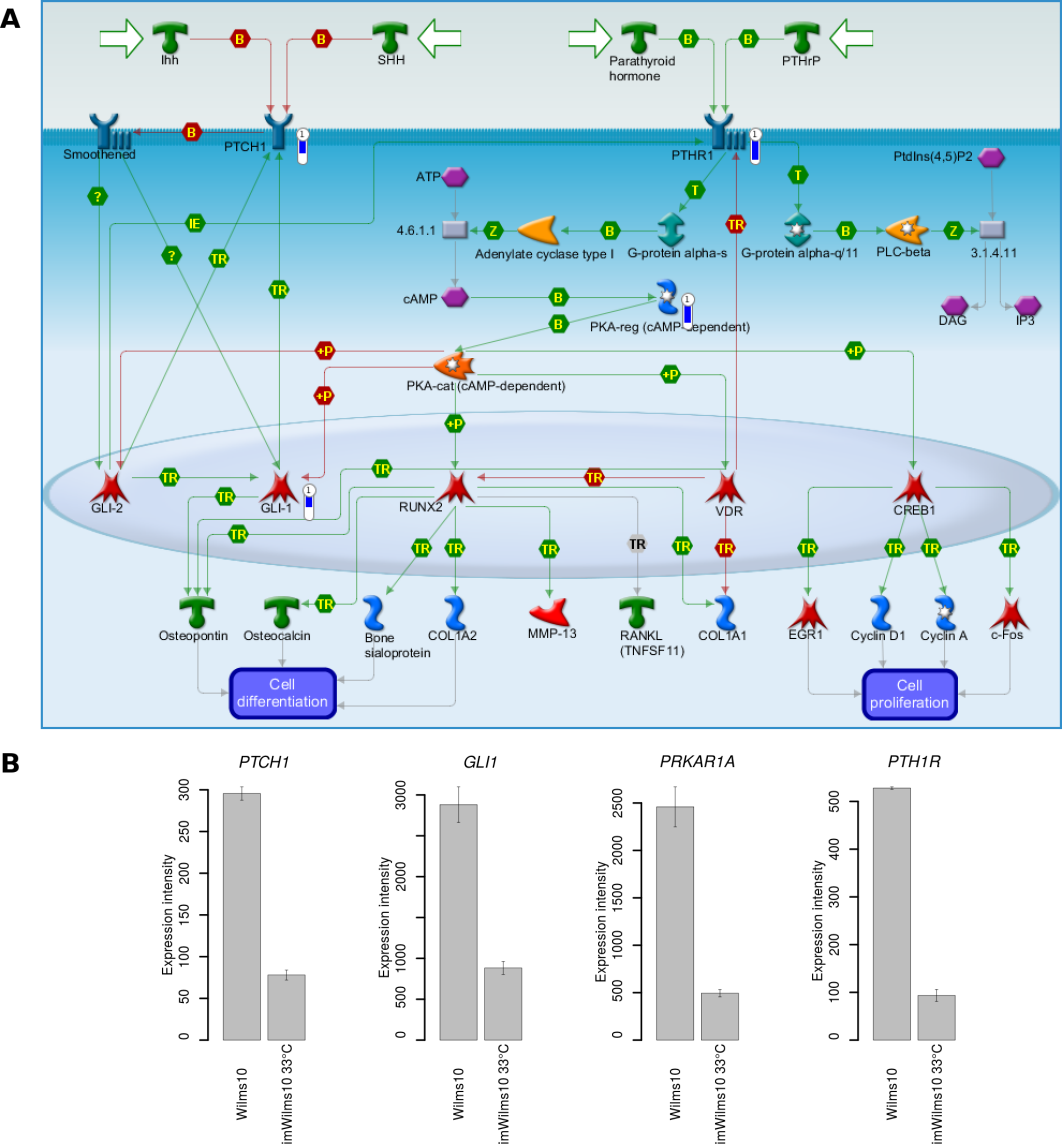
The top pathway down-regulated in imWilms10 versus non immortalized Wilms10 cells. (A) Using a gene set derived from the stringent parameters (FDR adjusted p-value of 0.1) the top down-regulated pathway in imWilms10 is Development_Hedgehog and PTH signalling pathways in bone and cartilage development". Down-regulated genes from this pathway are labelled with a thermometer. The height of the blue colour in the thermometer shows the fold down-regulation in the immortalized cells. (B) shown is the down-regulation of the four genes mapping to this pathway by expression intensity on the biological replicates on the arrays.

**Fig 7:**
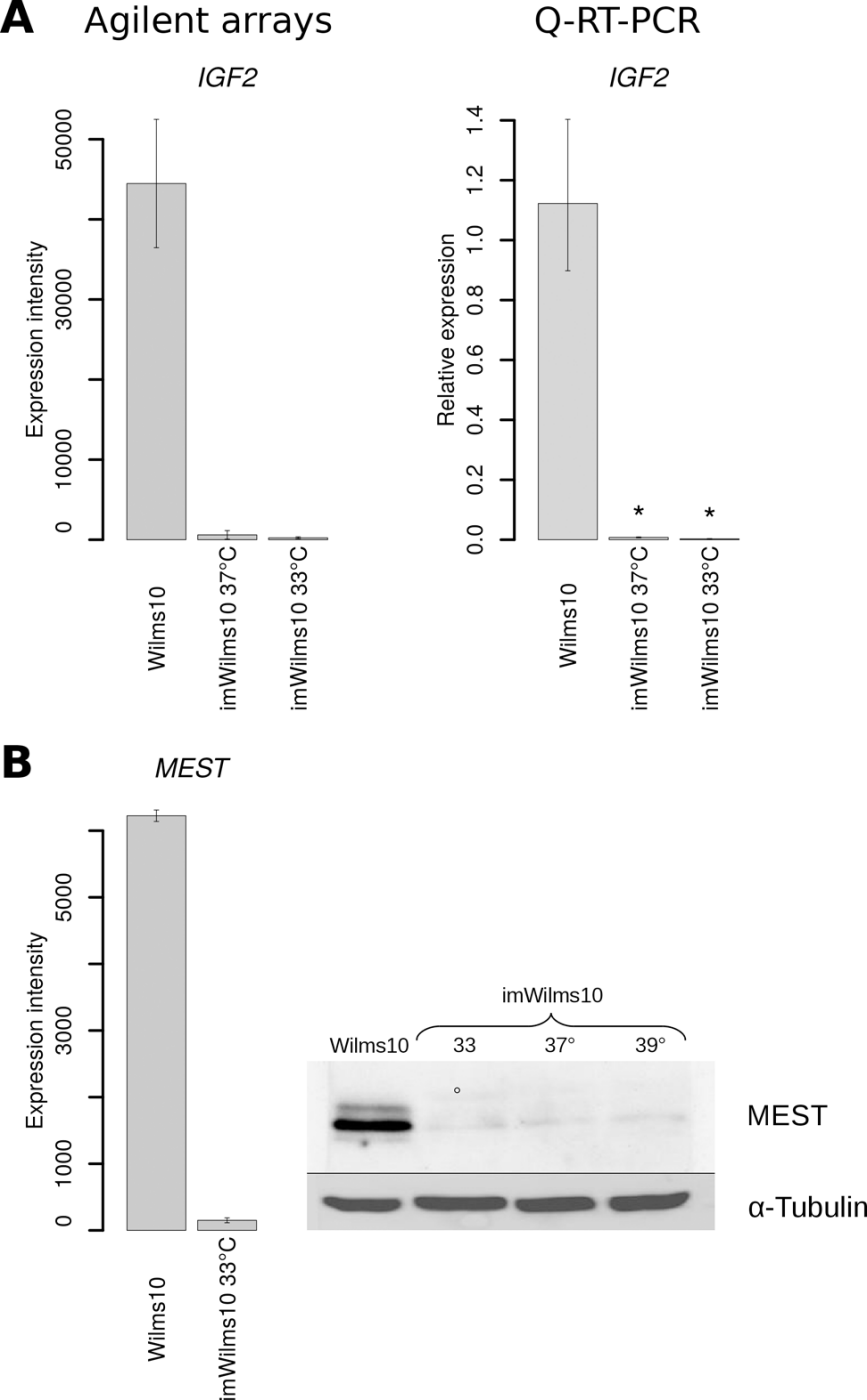
Down-regulation of the two embryonal growth factors *IFG2* and *MEST*. (A) Down-regulation of *IGF2* in imWilms10 cells cultured at 33°C as seen in two biological replicates on the Agilent array (left). The down-regulation was confirmed by Q-RT-PCR and is seen when cells are cultured at 33° and 37°C (right). The error bars correspond to 95% confidence intervals and * corresponds to a significance level of p 0.000001. (B) Down-regulation of *MEST* RNA as seen in the two biological replicates (left) and the confirmation of the protein down-regulation by western blot analysis. The down-regulation is seen at all temperatures, even at 39°C, the nonpermissive temperature for the tsLT, indicating that it is due to h*TERT* expression and is independent on the functional LT.

We next investigated the impact of the immortalization process by analyzing differentially expressed genes using Wilms10 cells as control. First, we analyzed differentially expressed genes using stringent parameters (FDR q < 0.1) and an absolute fold change > 1.5. Under these conditions we detected 270 genes with higher and 212 genes with lower expression levels in imWilms10 cells. To gain insight into the function of these genes we used the MetaCore algorithm “enrichment of pathway maps”. The 10 most significant pathway maps are shown in Fig 5. In the down-regulated gene set (Fig 5A) the pathway map “Development_Hedgehog and PTH signaling pathways in bone and cartilage development” is most significant. Fig 6 shows this pathway map and the down-regulated genes are indicated by blue bars. For example, from this pathway genes encoding *PTHR1* and *PTCH1* receptors as well as downstream genes *GLI1* and *PRKAR1A* (PKA-reg (cAMP-dependent)) are down-regulated in imWilms10 cells (Fig 6B). A complete list of >10 fold down-regulated genes is shown in S2 Table. We noted a down-regulation of *IGF2* (-214 fold, FDR p = 0.0141) and *MEST* (-45 fold, FDR p = 0.0117). The strong down-regulation of *IGF2* was confirmed by Q-RT-PCR (Fig 7A) and the down-regulation of *MEST* RNA and corresponding repression of the MEST protein is shown in Fig 7B. Other highly down-regulated genes encode transcription factors involved in early development, e.g. *SALL1*, *MAF*, *PBX4*, and *EYA2*. In the up-regulated gene set the "Retinol metabolism" and "Transcription_CoREST complex-mediated epigenetic gene silencing" are most significant (Fig 5B). A complete list of up-regulated genes (<10 fold) is shown in S3 Table.

When less stringent conditions were applied to gene expression analysis, fc > 1.5 and a raw p-value < 0.05, we identified significantly enriched pathway maps using the data set of up-regulated genes. These include “Cell cycle_Start of DNA replication in early S-phase” and “DNA damage_ATM/ATR regulation of G1/S checkpoint”. Here *Histone H1C*, *BARD1*, *CDC7*, *CCNE1*, *MCM3* and *CCNA1* gene expression levels were strongly induced in imWilms10 cells (S8 Fig). In this context it is important to note that LT interacts with p53 and pRB, enabling the cells to bypass a cell cycle control checkpoint (restriction point). This explains why cell proliferation proceeds even at later passages. However, the interpretation of these less stringent differential gene expression data should be viewed with caution. In summary the gene expression data confirm that imWilms10 cells have an extended proliferation capacity in comparison to Wilms10 cells.

MetaCore analysis using the data set of down-regulated genes revealed an enrichment of pathway maps and the ten most significant maps are shown in S9 Fig. For example the pathway map “Development-Regulation of epithelial to mesenchymal transition (EMT)” is highly significant. Several down-regulated genes of this pathway encode receptors for example *PDGFRA*, *PDGFRB*, *TGFBR1*, *TGFBR2*, *EDNRA* and *EDNRB*. In addition, the expression of genes encoding receptor ligands and downstream signaling components are down-regulated in imWilms10 cells. These include *BMP2* and *BMP4* (S10A Fig) and *WNT16*, *ERK1*/2, *SMAD4*, *MAPK14*, *RPS6KA2*, *SNAI2*, *ZEB2* and *TWIST1*. Another important result is the down-regulation of several genes from the Wnt signaling pathway. These genes predominantly encode negative regulators of Wnt signaling e.g. *AXIN2*, *NKD2*, *SFRP2* and the transcriptional corepressor *TLE1* (S10B Fig). Furthermore, we detected strong down-regulation of *ROR1* and *ROR2* genes from the noncanonical Wnt signaling pathway.

**Fig 8:**
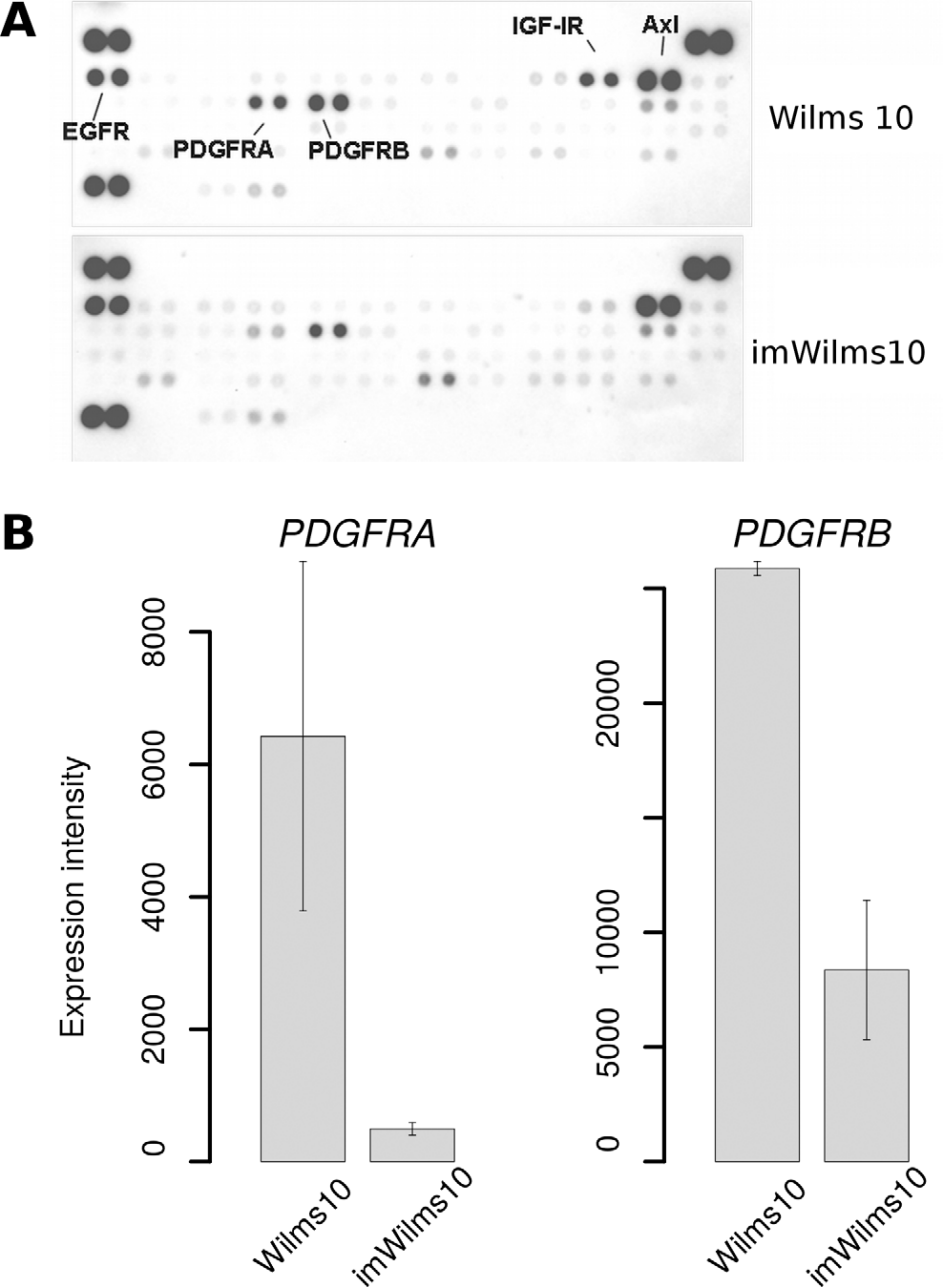
Human Phospho-RTK array blot and down-regulation of *PDGFRA* and *PDGFRB* genes. (A) Protein extracts from Wilms10 and imWilms10 cells cultured at 33°C were analyzed for the phosphorylation status of 49 receptors using the ProteomeProfiler^TM^ Phospho-RTK Array kit. Each receptor is spotted in duplicates. The top blot represents protein analysis of parental Wilms10 cells and the position for the highly phosphorylated receptors are labelled with their names. Below, the analysis of protein extracts from the imWilms10 cells. A reduction in the phosphorylation IGF-1R, PDGFRA and PDGFRB receptors is seen, whereas Axl and EGFR remain unchanged. The strong signals in the left top row, right top row and bottom left row are positive control spots, indicating that the same amount of protein extracts were analyzed. (B) Expression intensity of *PDGFRA* and *PDGFRB* genes in Wilms10 cells and imWilms10 cultured at 33°C. The error bar represents the standard error derived from the two biological replicates on the array.

To study whether the down-regulation of genes encoding receptors has functional consequences we investigated their phosphorylation status using human Phospho-RTK-proteome profiler arrays. Here phosphorylation levels of 49 receptors can be detected simultaneously. The results are shown in Fig 8A. Both, PDGFRA and PDGFRB show reduced phosphorylation levels in imWilms10 cells. In addition, IGF-IR phosphorylation is reduced to background level, suggesting that this receptor is inactive. The phosphorylation status of other receptors, e.g. Axl and EGFR, is not affected by immortalization. The reduced phosphorylation levels of PDGFRA and PDGFRB correlate with down-regulation of the corresponding genes in the immortalized cells (Fig 8B). In contrast, *IGF-1R* mRNA expression was not altered (S11 Fig), while IGF-1R receptor phosphorylation levels were drastically reduced in imWilms10 cells. In this context it is interesting that *IGF2*, one of the ligands for IGF-1R, is the second most down-regulated gene in imWilms10 cells (Fig 7A). Thus the absence of IGF2 might be linked to low IGF-1R phosphorylation levels in imWilms10 cells.

In summary, our gene expression data show that parental and imWilms10 cells express specific kidney marker genes at similar levels. In addition Wilms10 cells express genes of diverse differentiation programs simultaneously, similar to human mesenchymal stem cells (hMSC). A similarity of Wilms tumor derived cell lines with mutant *WT1* to hMSCs was previously reported by our group [18]. Furthermore, our proteome profiler data show that diverse signalling pathways are active in parental Wilms 10 cells that are efficiently down-regulated during the process of cell immortalization, suggesting that immortalization is associated with growth factor independence, an important feature of highly malignant cancer cells.

### Differentiation potential of parental Wilms10 and imWilms10 cells

**Fig 9:**
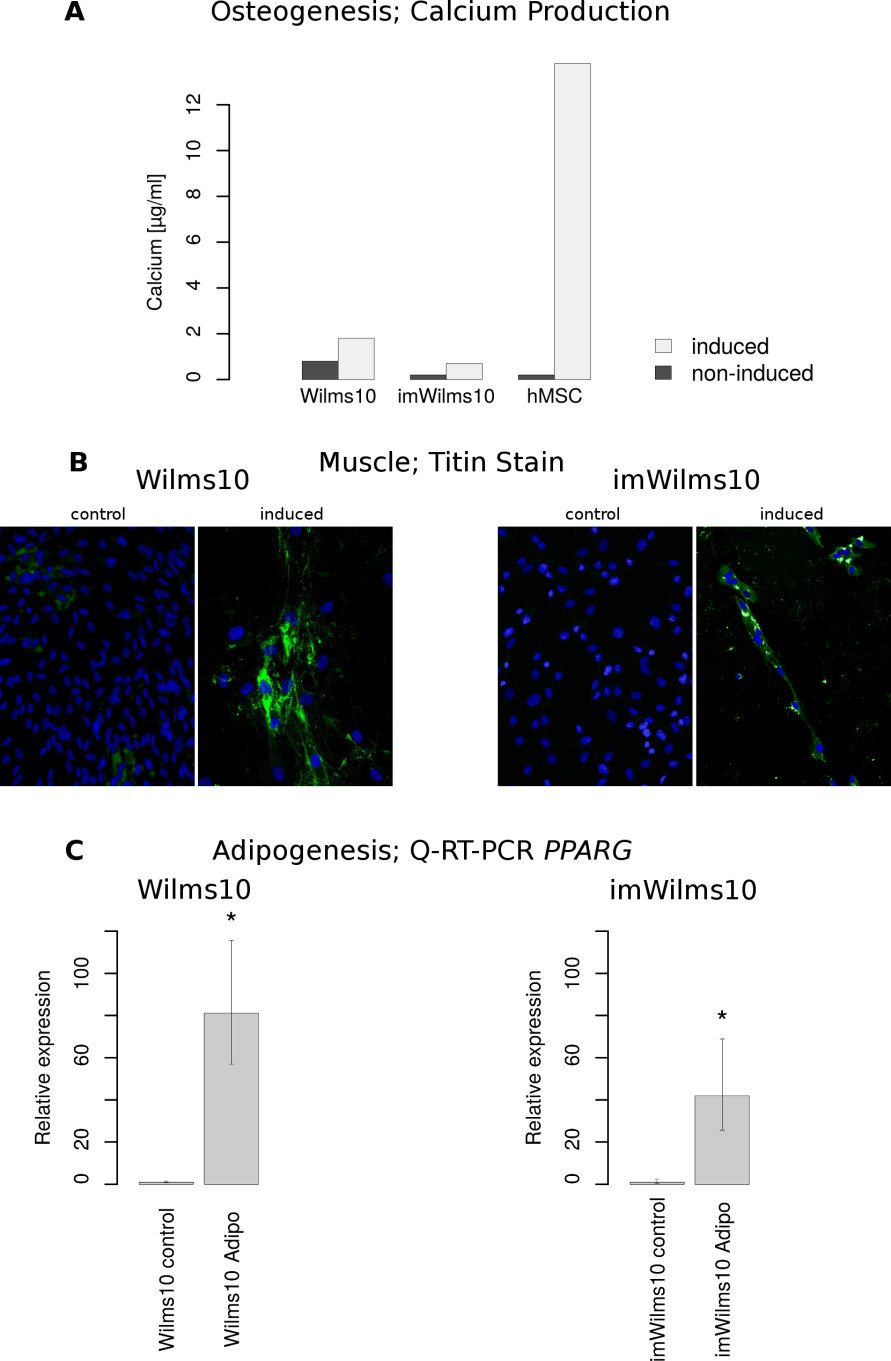
Differentiation potential of Wilms10 and imWilms10 cell lines. (A) Analysis of osteogenesis as measured by quantification of calcium production. Parental Wilms10 cells deposit some calcium in the absence of induction conditions. hMSC cells were used as controls and they show a high level of calcium production after induction of differentiation. The imWilms10 cells, demonstrate a modest increase of calcium production as compared to parental Wilms10 cells. (B) Analysis of muscle differentiation by immunofluorescence analysis using a Titin antibody. Left: Most Wilms10 cells show positive staining for Titin after 9 days of induction. Right: the same analysis was performed with imWilms10 cells. A lower percentage of cells showed a positive staining for Titin. (C) Quantitative analysis *PPARG* mRNA expression, as a marker for adipogenesis. Left: After 10 days of induction a significant increase is seen in Wiilms10 cells compared to the uninduced control. The expression was normalized versus *RER1* and the analysis was done in triplicates. The error bar corresponds to 95% confidence intervals and * corresponds to a significance level of p = 0.00001- Right: The same analysis was conducted with imWilms10 cells and a slightly lower induction was observed when compared to uninduced control cells. Expression analysis was done after 18 days of induction of imWilms10 cells. The error bar corresponds to 95% confidence intervals and * corresponds to a significance level of p = 0.00001.

A number of genes involved in osteogenic, adipogenic and muscle differentiation are expressed at a low level in Wilms10 cells. Therefore, we tested whether parental Wilms10 cells as all other *WT1* mutant cells have a differentiation potential for these cell fates. Osteogenic differentiation was analyzed by Alizarin red staining and Wilms10 (S12A and S12B Fig) and imWilms10 cells were negative. The control hMSC cells were positive (S12C and S12D Fig). Therefore in the next experiment osteogenic differentiation was measured by calcium deposits as shown in Fig 9A. After 19 days in culture, Wilms10 cells show low levels of calcium compared to hMSC. Note that uninduced Wilms10 cells also deposit low levels of calcium. The analysis of imWilms10 in the same experiment and cultured at 37°C, where the tsLT should be partially inactivated, showed an even lower potential for osteogenic differentiation (Fig 9A). This is not surprising, as the differentiation process is normally associated with a loss of growth potential. However, imWilms10 continue to proliferate when differentiation programs are induced. Moreover, the induction of osteogenic differentiation was not associated with up-regulation of two genes involved in osteogenesis, *BGLAP* and *ALPL*, as measured by Q-RT-PCR after 5 and 10 days of induction (not shown). This indicates that only a few cells have reached a more terminal osteogenic differentiation state and deposit calcium.

To analyze the cells for muscle differentiation we used immunofluorescence with a Titin antibody. Titin is only produced in different muscle cells [30]. Expression of Titin was seen in 24% of the Wilms 10 cells after 9 days of induction (Fig 9B). In the control culture of human skeletal myoblasts (HSMM) 24% of the cells were positive for Titin (not shown). The imWilms10 cells also showed induction of the Titin protein in 10% of the cells (Fig 9B). A few multinucleated cells were observed in Wilms10 cells by phase contrast already 3 days after muscle induction (S13 Fig).

Staining of Wilms10 cells with Oil red O after 3 weeks of adipogenic differentiation was negative (S14A and S14B Fig), whereas control hMSC showed many stained lipid droplets (S14C and S14D Fig). However, Wilms10 cells acquired a more flattened/cuboidal shape, and their growth was inhibited (S14E Fig). As Oil red O stains lipid droplets in cells that have reached a terminal differentiation stage, we analyzed whether the cells are able to express some markers of adipogenic differentiation. We therefore measured the expression of two adipogenic marker genes, *LPL* and *PPARG* [24] by Q-RT-PCR after 0 and 10 days in adipogenic differentiation medium. A significant increase in the expression of *PPARG* but not *LPL* in comparison to uninduced control cells was observed in Wilms10 cells, indicating a limited adipogenic differentiation potential (Fig 9C). The analysis of imWilms10 cells also revealed a significant increase of *PPARG* expression in comparison to uninduced cells albeit at lower levels than in parental cells (Fig 9C).

Although for differentiation experiments imWilms10 were cultured at 37°C, their growth rate was not reduced. The continuous proliferation of imWilms10 cells most likely counteracts terminal differentiation in our experiments. To evaluate whether imWilms10 cell proliferation depends on a functional LT, we determined their colony forming ability and their growth potential at 33°C (permissive temperature), 37°C (semipermissive temperature) and 39°C (nonpermissive temperature). The imWilms10 cells formed large colonies at 37° and 39°C with an efficiency of 77.2% and 80.7% respectively, whereas at 33°C only 39.3% of the cells formed small colonies (S15 Fig). Similar results were obtained by determining the population doubling time of cells cultured at the three temperatures after 11 days in culture. At 33°C the doubling time was 120h, at 37°C 63h and at 39°C 64h. These analyses suggest that the expression of a functional LT is not necessary for the continuous growth of imWilms 10 cells.

Our differentiation experiments showed that the parental Wilms10 cells have restricted potentials for muscle, osteogenic and adipogenic differentiation, while these differentiation potentials are still present in imWilms10, they are even further reduced.

## Discussion

We describe here a Wilms tumor (Wilms10) with an unusual tumor specific homozygous deletion of *WT1* nested within a heterozygous 11p13 deletion. The 228kb homozygous deletion only covers the *WT1* gene and the 1Mb heterozygous 11p13 deletion extends from *WT1* to *HIPK3*. In addition, UPD restricted to 11p15.2pter and including the *IGF2* gene was identified, suggesting a more distal mitotic recombination in 11p15. The high expression of *IGF2* in these cells indicates that the paternal allele was duplicated, whereas the rest of chromosome 11 was derived from both parental alleles. We have thus identified a Wilms tumor with a complete lack of *WT1* due to a homozygous deletion. It is highly desirable to establish tumor derived cell lines to develop cell culture model systems for Wilms tumors with deleted *WT1* genes. Thus our immortalized cell line (imWilms10) represents the first cell culture model system for Wilms tumors with deleted *WT1* genes. The immortalized imWilms10 cells were characterized by the expression of selected kidney marker genes, by gene expression profiling, DNA analysis and cell differentiation experiments and were compared to parental Wilms10 cells.

We have previously established a number of cell lines that were derived from Wilms tumors with *WT1* mutations. These cell lines harbor two genomic DNA copies of mutant *WT1*, and all of them have two copies of chromosome 11 (unpublished observation). In most cases, the homozygous mutation of *WT1* is due to a mitotic recombination event between the last heterozygous marker in 11q11 and the first homozygous marker in 11p12 (unpublished observation). The high expression of *IGF2* and low expression of *H19* in these cell lines indicates that the paternal allele is duplicated (unpublished observation, BR-P). In one cell line from a WAGR case with a 11p13 deletion on one allele, the remaining copy of *WT1* carries a mutation, i.e. the *WT1* mutation is hemizygous; both paternal chromosome 11 alleles are present in this case except for the 11p13 deletion. These cell lines express different levels of mutant *WT1* mRNAs, whereas Wilms10 cells do not express any *WT1* mRNA (see Fig 2). This is an important difference as mutant WT1 proteins exhibit gain of function properties [10].

Cultured Wilms10 cells harbored a homozygous p.S45Δ *CTNNB1* mutation due to UPD on chromosome 3p21pter including *CTNNB1*. This mutation is different from the one detected in the primary Wilms10 tumor. The presence of different *CTNNB1* mutations supports the sequential linear model whereby initiating mutations first occur in *WT1*, and for tumor development, additional *CTNNB1* mutations are needed. By microdissection of tumor material and ILNR lesions it has been observed that various different *CTNNB1* mutations occur in *WT1* mutant cells, an example of genetic heterogeneity in Wilms tumor [7, 9] (unpublished observation Uschkereit and BR-P).

Immortalized imWilms10 cells have a stable karyotype and could be propagated at 33° for extended periods of time and thus can be considered a bona fide cell line. At this stage we have cultivated imWilms10 cells for 30 passages, corresponding to 90 population doublings. However, imWilms10 cells also proliferated at 37°C and even at 39°C. The shift of imWilms10 cells to the semipermissive temperature 37°C, should result in a partial functional inactivation of the tsLT. Our gene expression profiling experiments clearly showed that a larger number of genes are differentially expressed between Wilms10 and imWilms10 cells when these are cultured at the permissive temperature than at the semipermissive temperature (data not shown). This indicates that the tsLT is indeed functionally temperature sensitive. The complete inactivation of the tsLT occurs at 39°C, but we did not study the cells further at that non-physiological temperature. However, a low level of the tsLT protein was detected by Western blot analysis of imWilms10 cells cultured at 39°C, suggesting that inactivation of the tsLT protein is due to a conformational effect. Hardy et al., who studied normal human mammary fibroblasts immortalized with telomerase and a tsLT (HMF3A), also detected the tsLT protein at 37° and 39°C by Western blotting [31]. It is interesting that imWilms10 cells continue to grow at 37°C, whereas, the HMF3A cells and other normal immortalized cells senesce, when the tsLT is inactivated [12–17, 31]. We have observed that normal hMSC immortalized with telomerase and the same tsLT construct did not grow well at 37°C. After prolonged culturing at 39°C, they showed abnormal muscle differentiation and stopped their growth completely (Görlt and BR-P, unpublished). Two other immortalized Wilms tumor derived cell lines also continued to grow at the semipermissive temperature of 37°C (AB, Tenbusch and BR-P, unpublished). This suggests that LT is needed for the immortalization process but established immortalized Wilms tumor cell lines no longer depend on a functional LT. This difference to “normal cells” where expression of LT is needed to keep the cells in an immortalized state is likely due to the fact that these are tumor cells.

In order to study whether the immortalized cells retain biological properties of the parental Wilms10 cells we first studied the expression of several characteristic kidney marker genes. Laser capture microdissection of major kidney compartments at mouse embryonic day E15.5 and fluorescence-activated cell sorting of component-specific GFP transgenic mice established a kidney atlas of gene expression [28]. *FoxD1* is highly expressed in cortical and nephrogenic interstitium, in the same compartment that expresses *Meis1* [28]. Expression of Pax3 in metanephric mesenchyme and in the stromal compartment of the developing mouse kidney was recently demonstrated by immunohistochemistry [29]. *Osr1* was shown to identify multi-potent cells of intermediate mesoderm and its expression becomes restricted during kidney development to an *Osr1*-dependent nephron progenitor compartment [32]. The related gene, *Osr2*, was also shown to be expressed during kidney development [33]. The *Six1* gene plays a role in limb development and in the developing kidney; it is expressed in uninduced metanephric mesenchyme at E10.5 and in induced mesenchyme at E11.5 [34]. Furthermore, SIX1 protein expression was shown in the blastemal elements of Wilms tumors [35]. These characteristic genes are expressed in all *WT1* mutant cell lines and Wilms10 cells and are unaltered in imWilms10 cells. This indicates that these *WT1* mutant cells may correspond to an early stage of kidney development before specification of the different lineages and the imWilms10 cells have retained these properties. In addition other genes expressed in kidney cells such as *SNPO*, *CD2AP* and *NES* are not changed by the immortalization process.

One major difference between the Wilms10 cells and imWilms10 cells is the up-regulation of cell cycle genes caused by the expression of h*TERT* and the tsLT. Another difference is their state of signaling pathways. Paternal Wilms10 cells showed activation of several signaling pathways, i.e. PDGFR, EGFR, IGF and AXL as revealed by proteome profiler antibody arrays. Upon immortalization, these cells show a down-regulation as well as functional inactivation of several enzyme-linked receptors (RTK). In the case of PDGFRA and PDGFRB decreased phosphorylation is linked to a down-regulation of mRNA expression. In contrast, down-regulation of IGF1R phosphorylation was observed with unchanged *IGF1R* gene expression levels. As phosphorylation of this receptor is triggered by ligand binding, the almost complete down-regulation of *IGF2* gene expression in imWilms10 cells might explain this finding. It is interesting that besides *IGF2*, expression of another fetal growth factor (*MEST*), is also reduced to basal levels after immortalization. The down-regulation of *IGF2* in imWilms10 cells is remarkable in the context of the paternal duplication of chromosome 11p15 and expression of the gene from both alleles. The down-regulation *IGF2* and *MEST* expression occurs at permissive and nonpermissive temperatures and is therefore independent of tsLT. It therefore likely that telomerase is linked to *IGF2* and *MEST* gene down-regulation in imWilms10 cells, as a function of telomerase in overall chromatin structure regulation and epigenetic mechanisms has been described [36]. Note that telomerase expression in bovine adrenocortical cells is correlated with an induction of *IGF2* expression [37], whereas it is associated with a repression of *IGF2* expression in immortalized Wilms tumor cells. This observation suggests that the effect of telomerase is highly cell context dependent and that the expression of fetal growth factors in embryonal Wilms tumor cells is not compatible with the establishment of immortalized cells with an unlimited life span. In summary, the immortalized Wilms tumor cells can proliferate despite a down-regulation of ligands and receptors for several signaling pathways. It will be of interest to study the mechanisms that lead to the activation of these different signaling pathways in the parental Wilms tumor cells and the effects of their down-regulation on the growth of these cells. This should be kept in mind when novel therapeutic approaches targeting of one of these signaling pathways are considered, as the others can still be activated [38]. However, a reduction of tumor growth was observed in mice treated with a specific inhibitor for IGF1R. In this case, the tumor was established by orthotopically injecting Wilms tumor cells with elevated IGF1R signaling but without a *WT1* mutation in the kidney of mice [39]. Therefore more basic experimental studies using different Wilms tumor cell lines are needed to develop individualized therapies targeting specific activated signaling pathways.

The primary Wilms10 tumor cells show a limited multilineage differentiation potential for osteogenesis, adipogenesis and muscle differentiation, similar to the other *WT1* mutant cells. The derived immortalized cells have retained these properties although at a reduced capacity, likely due to the fact that they continue to grow at 37°C, which is not compatible with differentiation. Therefore the parental Wilms tumor cell line with a novel and unusual chromosome 11 alteration and the immortalized derivative with a homozygous deletion of the entire *WT1* gene will be useful for further studies on the function of WT1 in Wilms tumor development, to further explore the origin of *WT1* mutant tumors as well as for the analysis of activated signaling pathways. Last not least these cells are valuable as negative controls to test various batches of WT1 antibodies, that are known for their unspecific cross reactivity with many proteins.

## Supporting Information

S1 Fig HE stain of a representative area of Wims10 and immunohistochemistry of TP53 in focal anaplasia. (PDF)

S2 Fig Two small heterozygous deletions on chromosome 1p. (PDF)

S3 Fig The size and position of the UDP in 11p15. (PDF)

S4 Fig The terminal 3p21pter UPD. (PDF)

S5 Fig Custom array with the exact extension of the deletion endpoints. (PDF)

S6 Fig Analysis of immortalized cells for T-antigen and h*TERT* expression. (PDF)

S7 Fig Enrichment analysis for up-regulated genes in imWilms10 cells. (PDF)

S8 Fig Comparison of the expression of up-regulated genes in the imWilm10 cells. (PDF)

S9 Fig Enrichment analysis for down-regulated genes in imWilms10 cells. (PDF)

S10 Fig Down-regulated genes. (PDF)

S11 Fig The *IGFR1R* gene expression is not down-regulated in imWilms10 cells. (PDF)

S12 Fig Osteogenic differentiation of hMSC and Wiilms10 cells. (PDF)

S13 Fig Muscle differentiation experiment with Wilms10 cells. (PDF)

S14 Fig Adipogenic differentiation experiment with hMSC and Wilms10 cells. (PDF)

S15 Fig Colony forming ability and population doubling time of imWilms10 cells cultured at 33°, 37° and 39°C. (PDF)

S1 Table Mutation status of WT cell lines and tumours. (PDF)

S2 Table Significantly down-regulated genes in imWilms10. (PDF)

S3 Table Significantly up-regulated genes in imWilms10. (PDF)
