# Supplementary material for: Establishment of a Conditionally Immortalized Wilms Tumor Cell Line with a Homozygous WT1 Deletion within a Heterozygous 11p13 Deletion and UPD Limited to 11p15
Source: PLoS One. 2016 May 23;11(5):e0155561. doi: 10.1371/journal.pone.0155561 (PMC4876997; doi:10.1371/journal.pone.0155561)
Supplement: S1 Fig — (PDF) [file pone.0155561.s001.pdf]

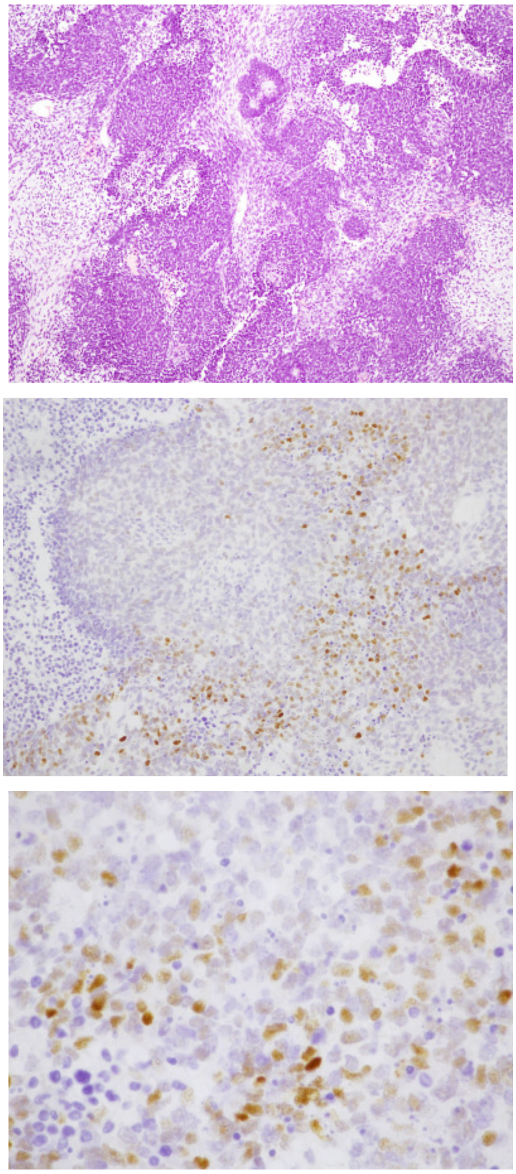

HE stain

p53 stain of focal anaplasia

enlargement of area with p53 positive cells

Figure S1. HE stain of a representative area of Wims10 and immunohistochemistry of TP53 in focal anaplasia
