# Supplementary material for: Establishment of a Conditionally Immortalized Wilms Tumor Cell Line with a Homozygous WT1 Deletion within a Heterozygous 11p13 Deletion and UPD Limited to 11p15
Source: PLoS One. 2016 May 23;11(5):e0155561. doi: 10.1371/journal.pone.0155561 (PMC4876997; doi:10.1371/journal.pone.0155561)
Supplement: S2 Fig — (PDF) [file pone.0155561.s002.pdf]

A

Heterozygous deletion 1p32

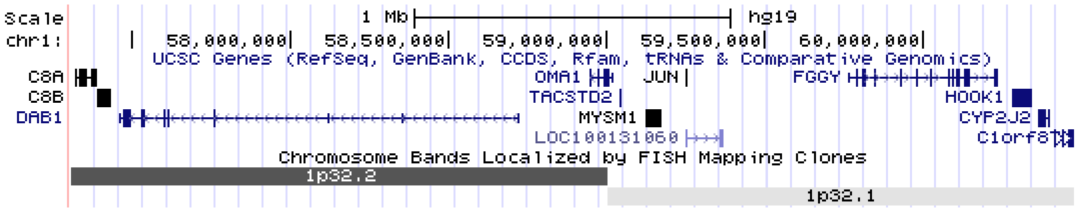

Heterozygous deletion 1p31.1

B

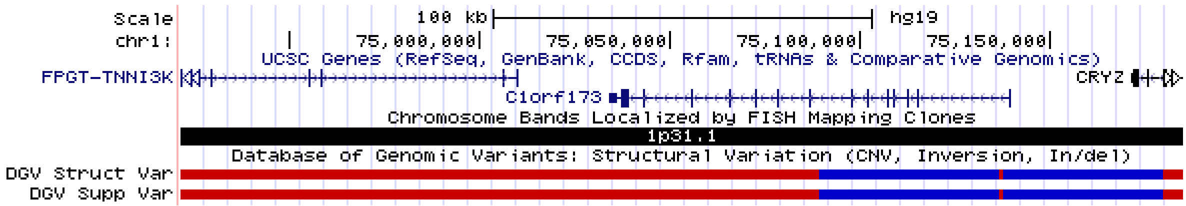

Figure S2. Two small heterozygous deletions on chromosome 1p
In aCGH/aSNP arrays two small heterozygous tumor specific deletions were detected. The exact position and extension is shown. **A**: the deletion in 1p32 covers several genes. The expression of neither of these genes is reduced in Wilms10 cells versus another WT cell line without a deletion. **B:** the smaller heterozygous deletion corresponds to a genomic segment that is listed as a structural variant, however, this deletion is not present in the germ line of the patient.
