# Supplementary figures and images for: Establishment of a Conditionally Immortalized Wilms Tumor Cell Line with a Homozygous WT1 Deletion within a Heterozygous 11p13 Deletion and UPD Limited to 11p15
Source: PLoS One. 2016 May 23;11(5):e0155561. doi: 10.1371/journal.pone.0155561 (PMC4876997; doi:10.1371/journal.pone.0155561)

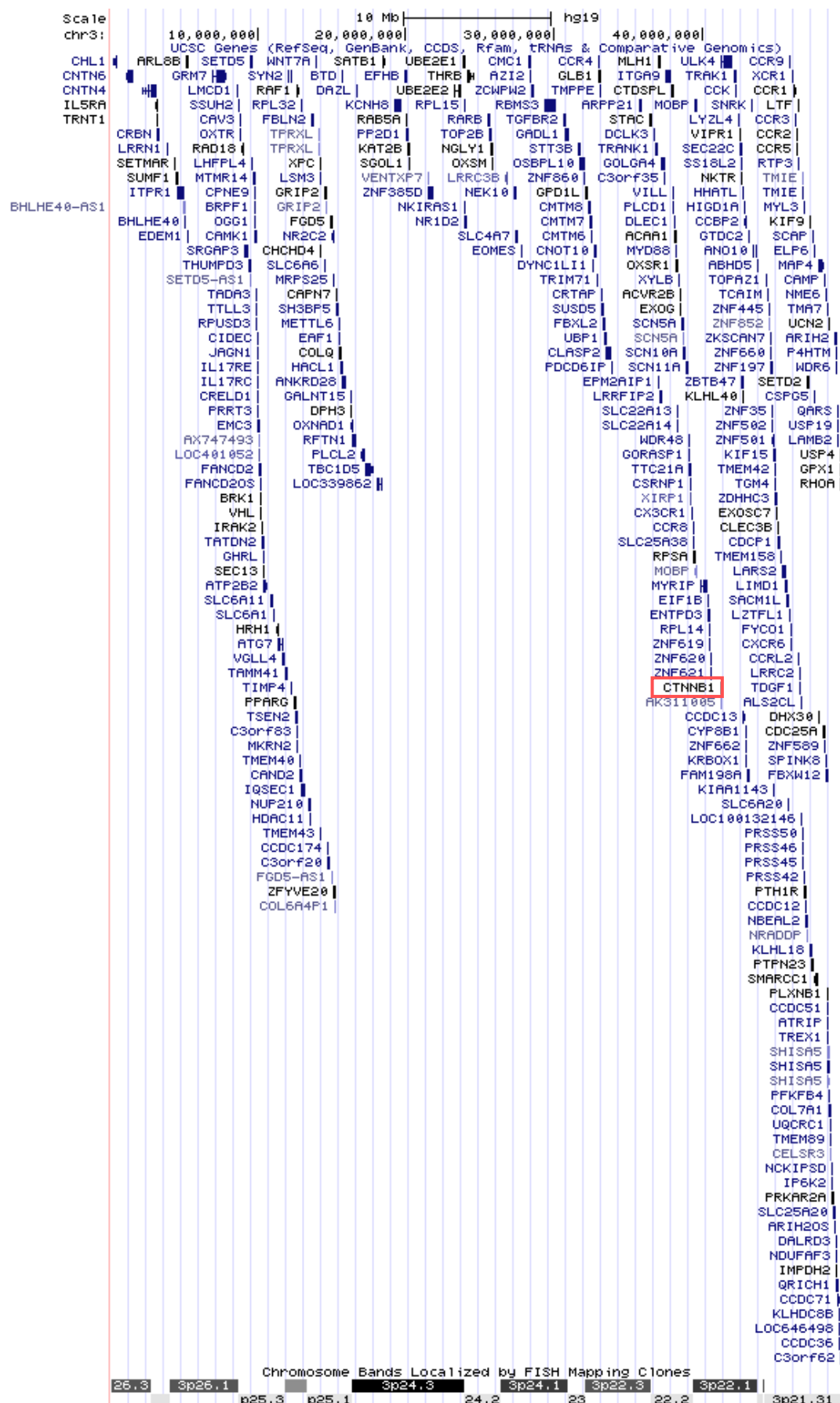

**Figure S4. The terminal 3p21pter UPD.**

The position of the *CTNNB1* gene is labelled with a red box.

Supplement: S4 Fig — (PDF) [file pone.0155561.s004.pdf]
