# Supplementary material for: Establishment of a Conditionally Immortalized Wilms Tumor Cell Line with a Homozygous WT1 Deletion within a Heterozygous 11p13 Deletion and UPD Limited to 11p15
Source: PLoS One. 2016 May 23;11(5):e0155561. doi: 10.1371/journal.pone.0155561 (PMC4876997; doi:10.1371/journal.pone.0155561)
Supplement: S5 Fig — (PDF) [file pone.0155561.s005.pdf]

tumor tumor cell culture

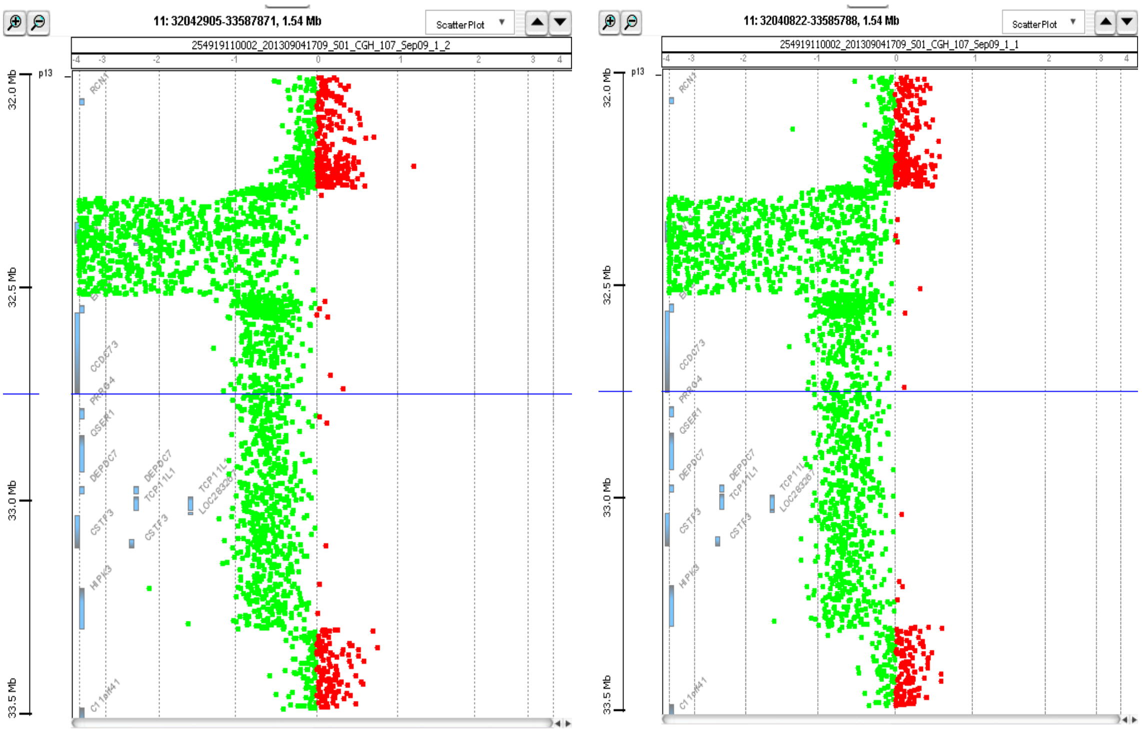

Chr.11	Tumor: position	
Deletion	Start	Stop
Heterozygous	32326435	32349847
Homozygous	32351497	32580825
Heterozygous	32581160	33377234

Chr.11	Tumor cell culture: position	
Deletion	Start	Stop
Heterozygous	32326435	32349847
Homozygous	32351497	32580825
Heterozygous	32581160	33377234

Figure S5. Custom array with the exact extension of the deletion endpoints

Top: The custom arrays of the tumor and tumor cell culture show the position of the homozygous and heterozygous deletion. The position of the *WT1* gene is completely covered with the oligonucleotides deleted and therefore it is not visible. Below: the exact positions of the deletion endpoints derived from the custom array for the tumor and the tumor cells culture is listed. The start and endpoints of the heterozygous deletion are shown in the first lane and in the second lane the position of the homozygous deletion within the heterozygous deletion is given.
