# Supplementary material for: Establishment of a Conditionally Immortalized Wilms Tumor Cell Line with a Homozygous WT1 Deletion within a Heterozygous 11p13 Deletion and UPD Limited to 11p15
Source: PLoS One. 2016 May 23;11(5):e0155561. doi: 10.1371/journal.pone.0155561 (PMC4876997; doi:10.1371/journal.pone.0155561)
Supplement: S6 Fig — (PDF) [file pone.0155561.s006.pdf]

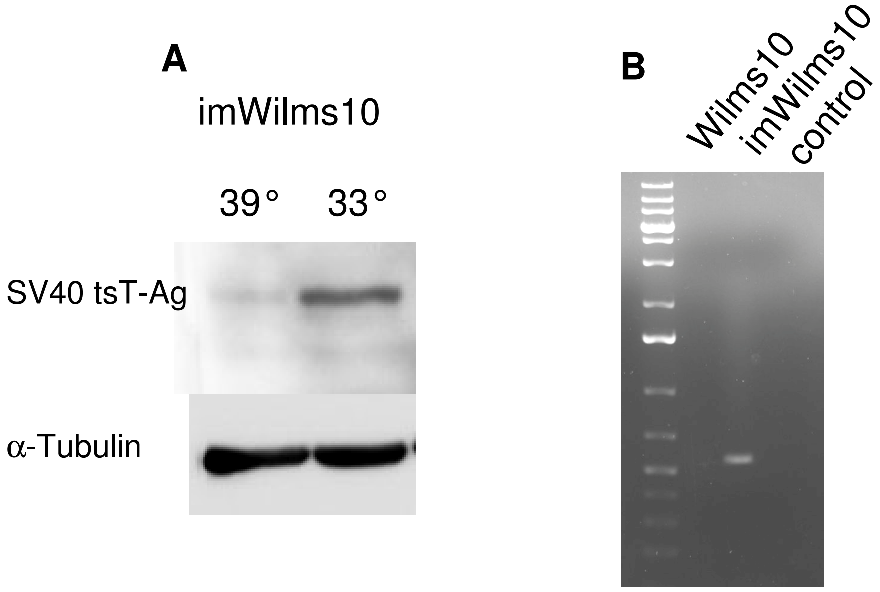

Figure S6. Analysis of immortalized cells for T-antigen and h*TERT* expression

A: T-Antigen expression in imWilms10 cultured at 33° and 39°C. **B**: The expression of the h*TERT* gene was analyzed by RT-PCR and is seen in the immortalized cells, whereas the original Wilms10 tumor cells do not express h*TERT*.
