# Supplementary material for: Establishment of a Conditionally Immortalized Wilms Tumor Cell Line with a Homozygous WT1 Deletion within a Heterozygous 11p13 Deletion and UPD Limited to 11p15
Source: PLoS One. 2016 May 23;11(5):e0155561. doi: 10.1371/journal.pone.0155561 (PMC4876997; doi:10.1371/journal.pone.0155561)
Supplement: S8 Fig — (PDF) [file pone.0155561.s008.pdf]

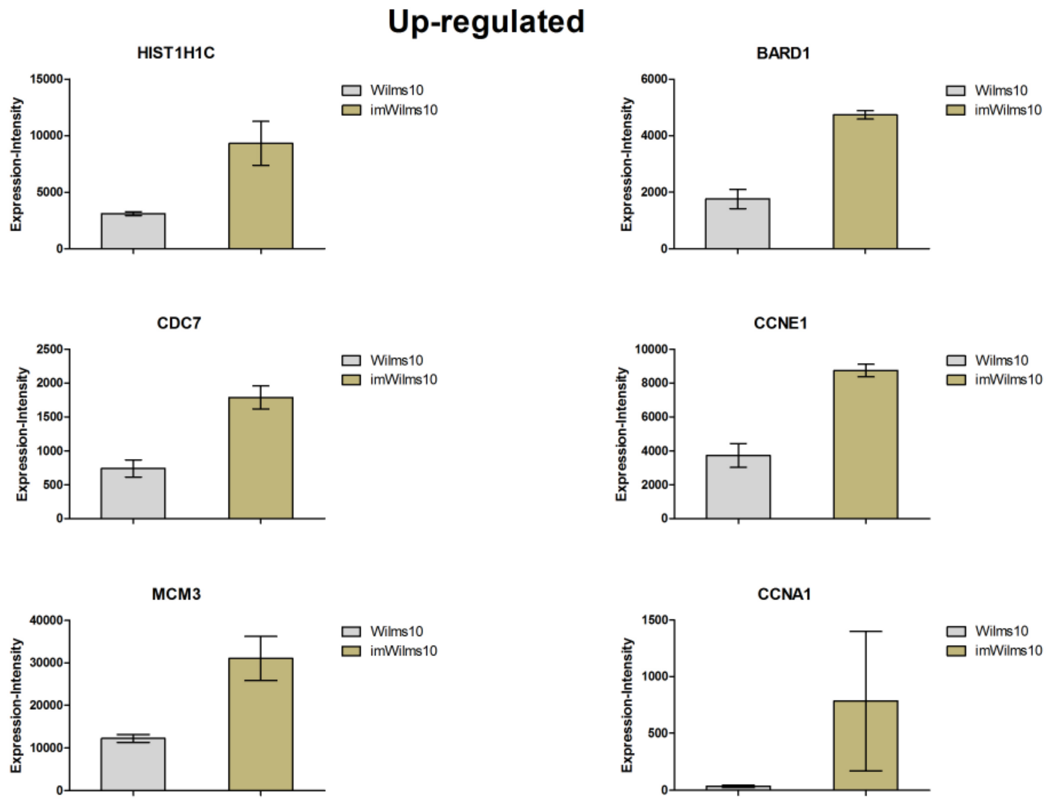

Figure S8. Comparison of the expression of up-regulated genes in the imWilm10 cells

This Figure shows the expression by intensity from the Agilent arrays of selected genes involved in cell cycle regulation. Error bar corresponds to standard error.
