# Supplementary material for: Establishment of a Conditionally Immortalized Wilms Tumor Cell Line with a Homozygous WT1 Deletion within a Heterozygous 11p13 Deletion and UPD Limited to 11p15
Source: PLoS One. 2016 May 23;11(5):e0155561. doi: 10.1371/journal.pone.0155561 (PMC4876997; doi:10.1371/journal.pone.0155561)
Supplement: S9 Fig — (PDF) [file pone.0155561.s009.pdf]

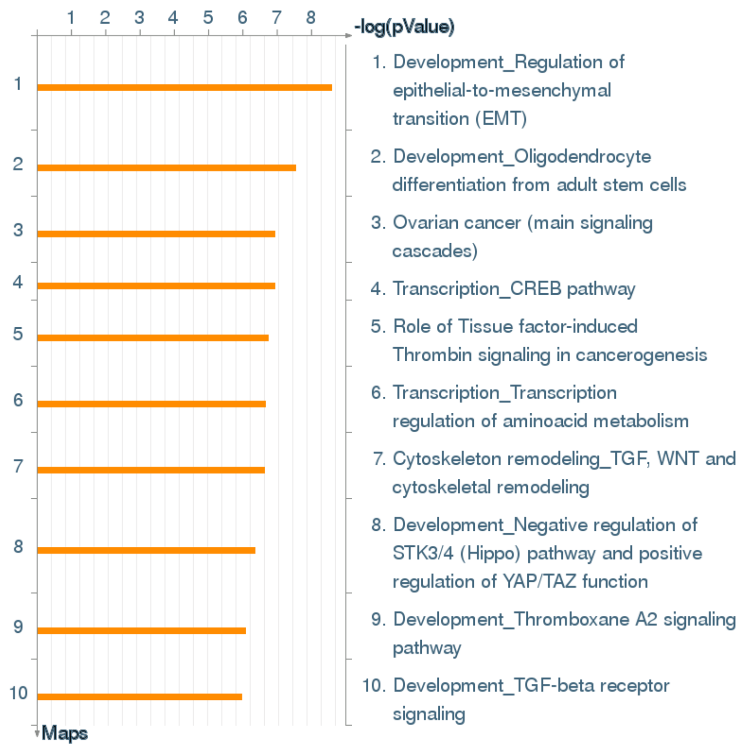


**Figure S9. Enrichment analysis for down-regulated genes in imWilms10 cells.**

The analysis was performed with genes down-regulated in imWilms10 cells cultured at 33° (fc>-1.5, p=0.05). This gene set was analyzed using the MetaCore enrichment pathway analysis. The 10 most signifcantly regulated pathways are shown here.
