# Supplementary material for: Establishment of a Conditionally Immortalized Wilms Tumor Cell Line with a Homozygous WT1 Deletion within a Heterozygous 11p13 Deletion and UPD Limited to 11p15
Source: PLoS One. 2016 May 23;11(5):e0155561. doi: 10.1371/journal.pone.0155561 (PMC4876997; doi:10.1371/journal.pone.0155561)
Supplement: S10 Fig — (PDF) [file pone.0155561.s010.pdf]

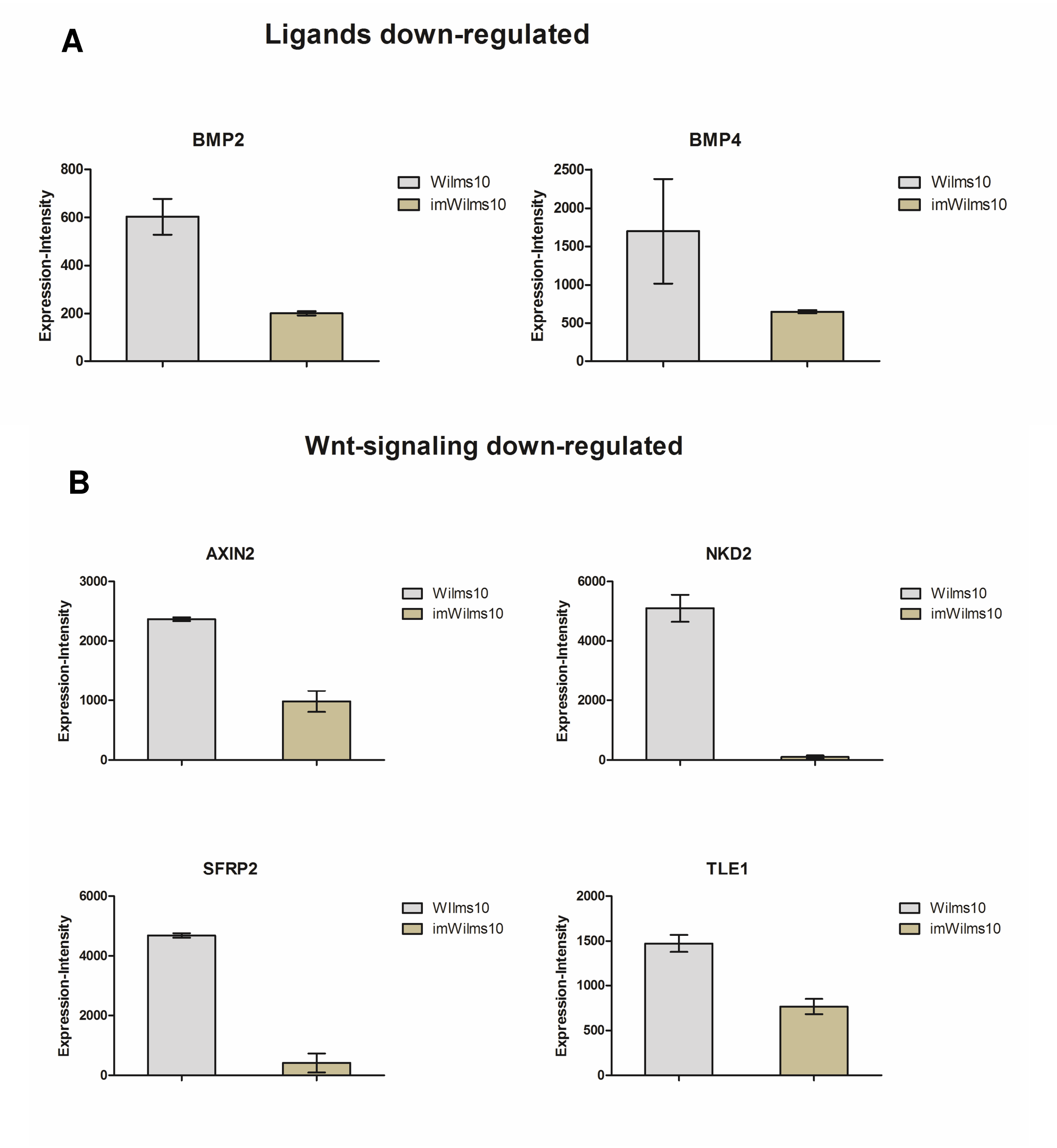

Figure S10. Down-regulated genes encoding ligands and Wnt signaling pathway genes in imWilm10 cells

This Figure shows the expression by intensity of Agilent array data of selected genes that are down-regulated in imWilms10 cells and are part of different signalling processes. Error bar represent the data of two arrays and corresponds to the standard error.
