# Supplementary material for: Establishment of a Conditionally Immortalized Wilms Tumor Cell Line with a Homozygous WT1 Deletion within a Heterozygous 11p13 Deletion and UPD Limited to 11p15
Source: PLoS One. 2016 May 23;11(5):e0155561. doi: 10.1371/journal.pone.0155561 (PMC4876997; doi:10.1371/journal.pone.0155561)
Supplement: S11 Fig — (PDF) [file pone.0155561.s011.pdf]

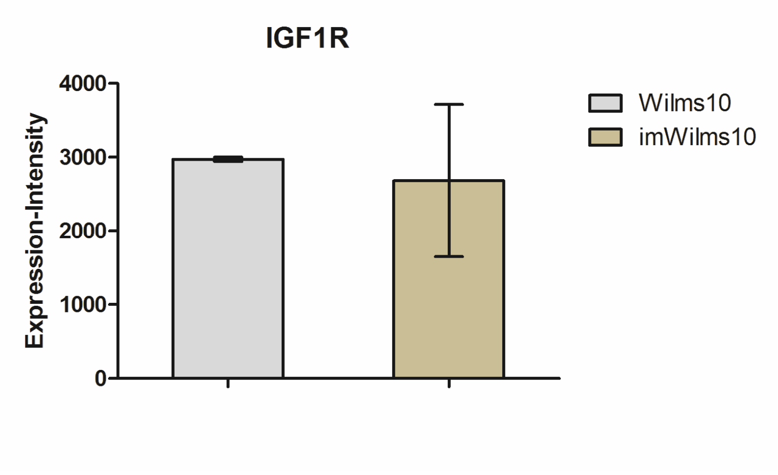

Figure S11. The *IGFR1R* gene expression is not down-regulated in imWilms10 cells

Expression intensity of *IGF1R* as determined by Agilent arrays, imWilms10 cells were cultured at 33℃.
