# Supplementary material for: Establishment of a Conditionally Immortalized Wilms Tumor Cell Line with a Homozygous WT1 Deletion within a Heterozygous 11p13 Deletion and UPD Limited to 11p15
Source: PLoS One. 2016 May 23;11(5):e0155561. doi: 10.1371/journal.pone.0155561 (PMC4876997; doi:10.1371/journal.pone.0155561)
Supplement: S12 Fig — (PDF) [file pone.0155561.s012.pdf]

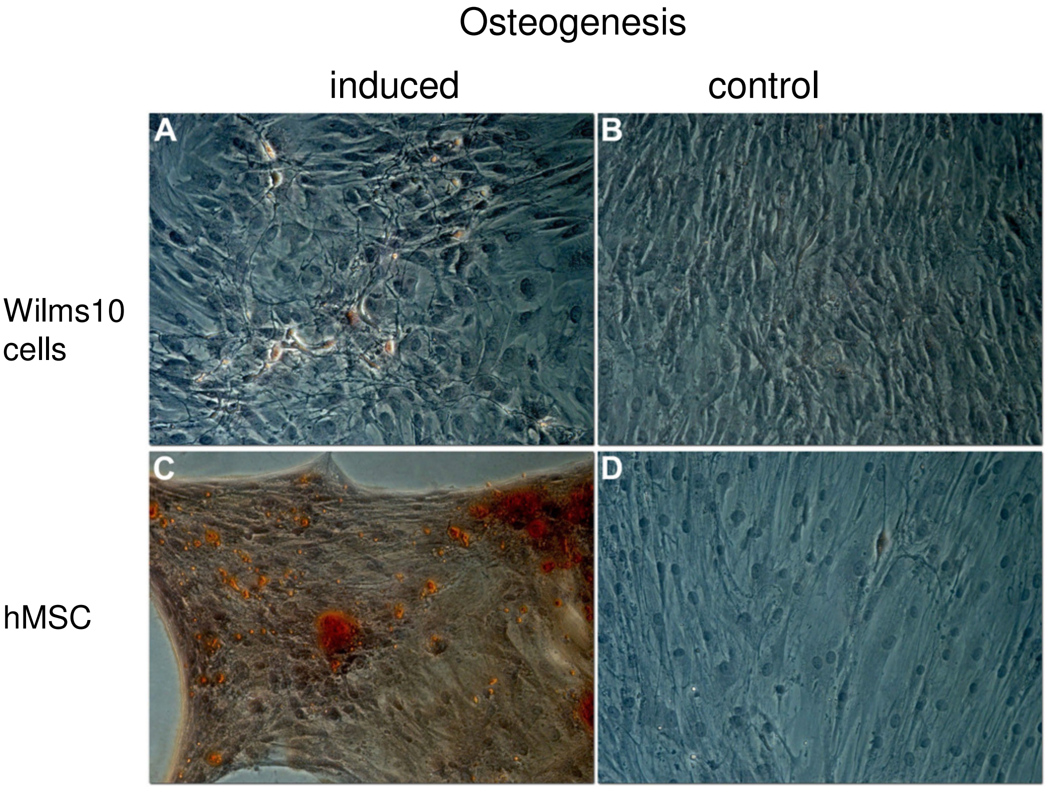

Figure S12. Osteogenic differentiation of hMSC and Wiilms10 cells
Wilms10 cells stained with Alizarin Red, (A) induced cells, (B) noninduced control cells. hMSC cells stained with Alzarin red, (C) induced and (D) noninduced control cells.
