# Supplementary material for: Establishment of a Conditionally Immortalized Wilms Tumor Cell Line with a Homozygous WT1 Deletion within a Heterozygous 11p13 Deletion and UPD Limited to 11p15
Source: PLoS One. 2016 May 23;11(5):e0155561. doi: 10.1371/journal.pone.0155561 (PMC4876997; doi:10.1371/journal.pone.0155561)
Supplement: S13 Fig — (PDF) [file pone.0155561.s013.pdf]

Muscle differentiation of Wilms10 cells

3 days

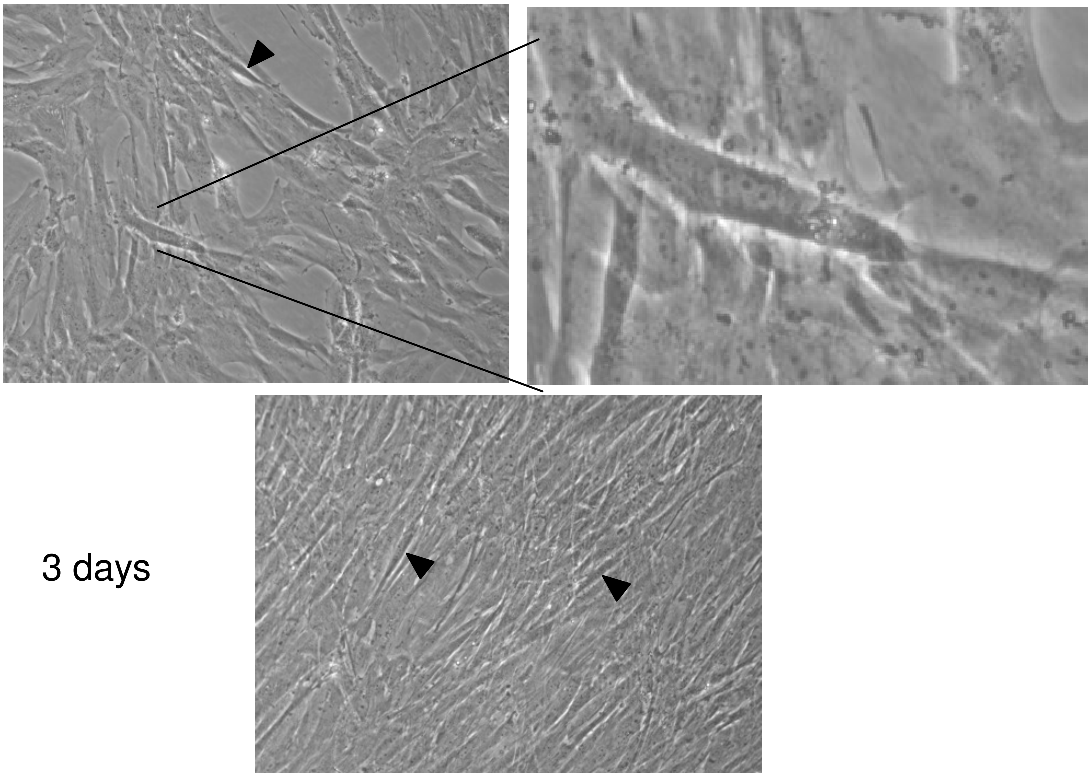

3 days

Figure S13. Muscle differentiation experiment with Wilms10 cells.

Individual multinucleated cells can be observed after 3 days of induction, indicating a more mature muscle stage. Enlargement of a multinucleated cell is seen on the right. In one observation field (below) there are only very few cells that are multinculeated but more bright shining cells with striation, typical for skeletal muscle cells can be observed (black arrow head).
