# Supplementary material for: Establishment of a Conditionally Immortalized Wilms Tumor Cell Line with a Homozygous WT1 Deletion within a Heterozygous 11p13 Deletion and UPD Limited to 11p15
Source: PLoS One. 2016 May 23;11(5):e0155561. doi: 10.1371/journal.pone.0155561 (PMC4876997; doi:10.1371/journal.pone.0155561)
Supplement: S14 Fig — (PDF) [file pone.0155561.s014.pdf]

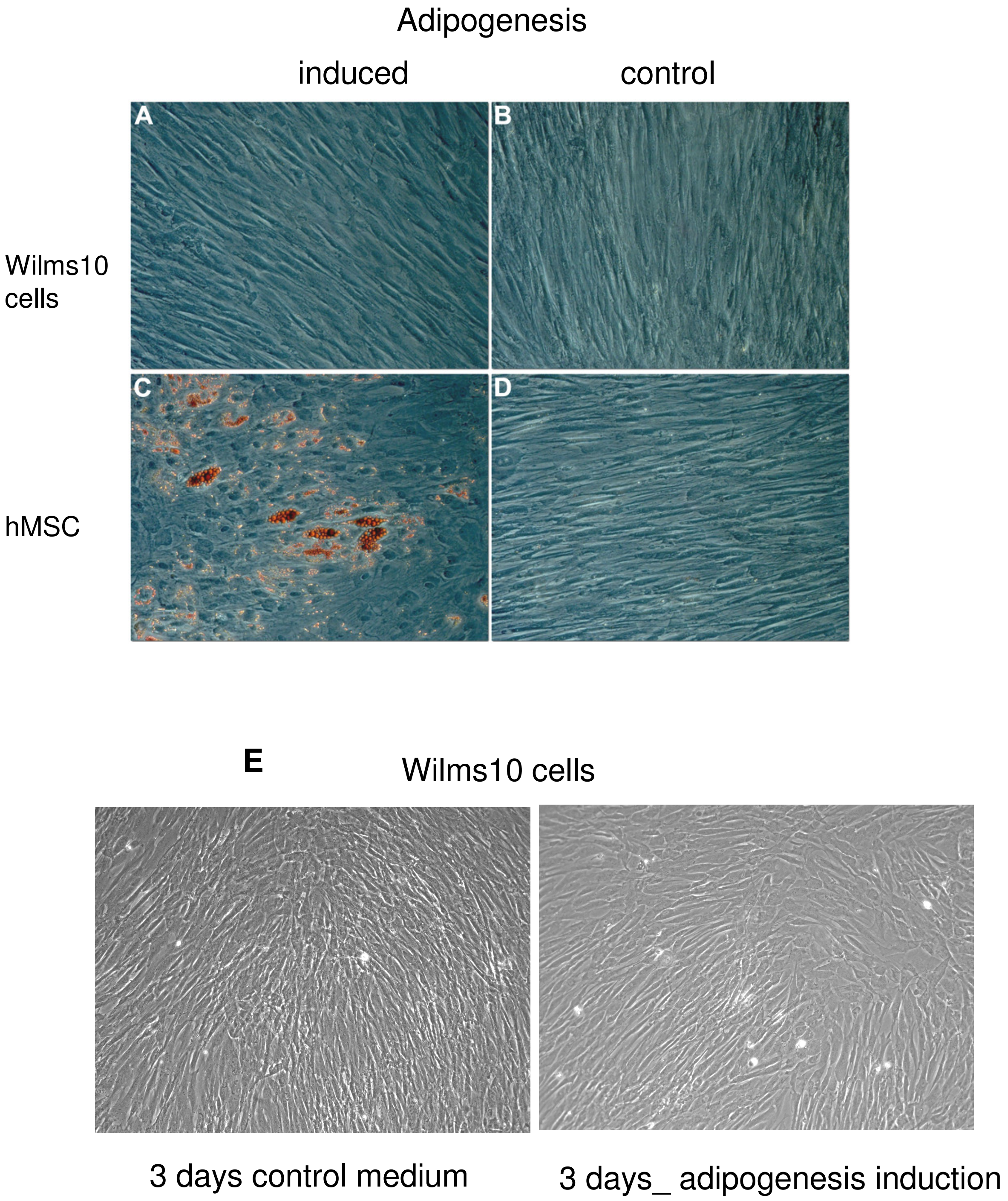

Figure S14. Adipogenic differentiation experiment with hMSC and Wilms10 cells.

Oil Red O stain of (A) Wilms10 cells induced, (B) noniduced control cells (C) hMSC induced and (D) noninduced control cells. (E) Change in morphology can be observed with Wilms10 cells to a more flattened phenotype in adipogenesis induction medium after 3 days (right).
