# Supplementary material for: Establishment of a Conditionally Immortalized Wilms Tumor Cell Line with a Homozygous WT1 Deletion within a Heterozygous 11p13 Deletion and UPD Limited to 11p15
Source: PLoS One. 2016 May 23;11(5):e0155561. doi: 10.1371/journal.pone.0155561 (PMC4876997; doi:10.1371/journal.pone.0155561)
Supplement: S15 Fig — (PDF) [file pone.0155561.s015.pdf]

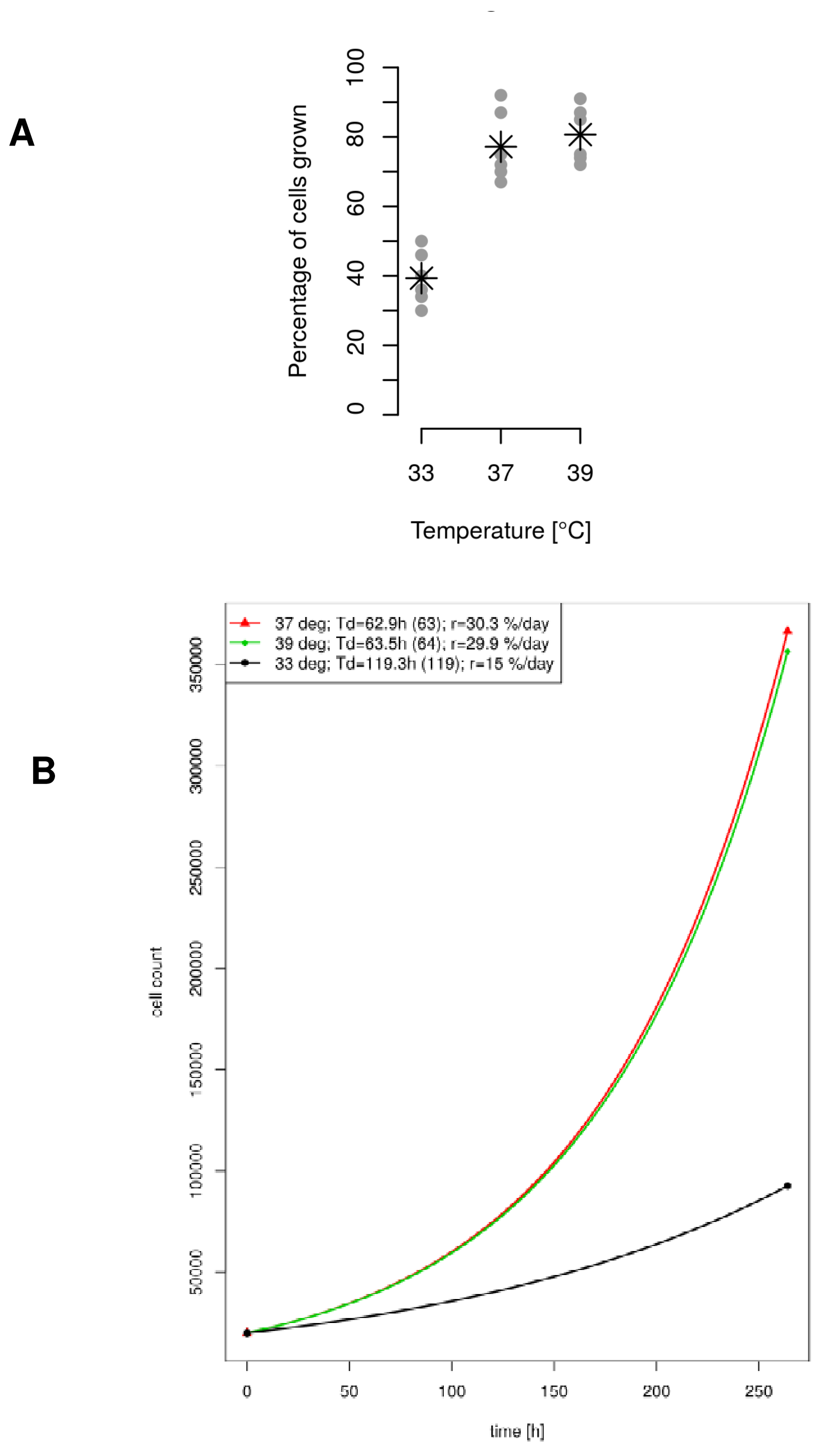

Figure S15. Colony forming ability and population doubling time of imWilms10 cells cultured at 33°, 37° and 39°C.

A: 100 cells were seeded per well in 6 well plates and cultured for 16 days. The colonies were stained and counted, the values correspond to number of colonies in each of the 6 wells in percent of the seeded cell number. The star indicates the mean of the counts in 6 wells. **B:** $1x10^4$ cells were seeded per well in 6 well plates and the cell number was determined after 11 days. The values are the mean of the cell count in 6 wells.
