# Supplementary material for: Establishment of a Conditionally Immortalized Wilms Tumor Cell Line with a Homozygous WT1 Deletion within a Heterozygous 11p13 Deletion and UPD Limited to 11p15
Source: PLoS One. 2016 May 23;11(5):e0155561. doi: 10.1371/journal.pone.0155561 (PMC4876997; doi:10.1371/journal.pone.0155561)
Supplement: S1 Table — (PDF) [file pone.0155561.s016.pdf]

Table S1. Mutation status of WT cell lines and tumours

WT1 mutation status of patient in blood DNA	*WT1*-mutation status in cell lines	LOH	*CTNNB1* mutation in cell lines
Wilms1 ,bilateral<br>c.149 C>A, S50X	homozygous p.S50X	11p11-11pter	heterozygous<br>TCT>TTT, S45F
Wilms2<br>c.1084C>T, p.R362X,	homozygous p.R362X	11p11-11pter	heterozygous<br>TCT>TAT, S45Y
Wilms3<br>wild type	Homozygous, c.1293-1294insA,<br>p.V432fsX87	11p11-11pter	wild type
Wilms4<br>WAGR, del 11p13	*WT1*-deletion and hemizygous<br>c.1311-1312insC, p.H438Pfs79	no LOH	heterozygous ΔS45
Wilms5<br>c.1168 C>T, p.R390X	homozygous, c.1296-1299delGC, p.R433Pfs84	11p11-11pter<br>loss of R390X	wild type
Wilms6 bilateral<br>c.1168C>T, R390X	homozygous<br>c.1168C>T, R390X	11p11-11pter	homozygous ΔS45
Wilms7<br>c.1180C>R<br>R394W (DDS)	heterozygous R394W,<br>cells were obtained after several passage in DMEM, likely only normal cells left	n.a.	n.a.
Wilms8<br>c.1168C>T, R390X	c.1168C>T, R390X	n. a.	p.S45A,<br>heterozygous
Wilms10<br>wild type	homozygous del WT1 and heterozygous del11p13	no LOH in 11p13 but UPD in 11p15	homozygous<br>ΔS45, UPD 3p
