# Supplementary material for: Establishment of a Conditionally Immortalized Wilms Tumor Cell Line with a Homozygous WT1 Deletion within a Heterozygous 11p13 Deletion and UPD Limited to 11p15
Source: PLoS One. 2016 May 23;11(5):e0155561. doi: 10.1371/journal.pone.0155561 (PMC4876997; doi:10.1371/journal.pone.0155561)
Supplement: S2 Table — (PDF) [file pone.0155561.s017.pdf]

GeneSymbol	Description	Wilms10_mean	imWilms10_mean	imWilms10_vs_Wilms10_mean.fc	imWilms10_vs_Wilms10.p	Combined.FDR
EPDR1	Homo sapiens ependymin related protein 1 (zebrafish) (EPDR1), transcript variant 1, mRNA [NM_017549]	4408	11	-388,72	0,001	0,077
CHST15	Homo sapiens carbohydrate (N-acetylgalactosamine 4-sulfate 6-O) sulfotransferase 15 (CHST15), transcript variant 1, mRNA [NM_015892]	1858	6	-311,65	0,001	0,065
KRT19	Homo sapiens keratin 19 (KRT19), mRNA [NM_002276]	43465	163	-266,18	0,000	0,040
IGF2	Homo sapiens insulin-like growth factor 2 (somatomedin A) (IGF2), transcript variant 1, mRNA [NM_000612]	43747	201	-218,24	0,000	0,062
GPC6	Homo sapiens glypican 6 (GPC6), mRNA [NM_005708]	1157	7	-155,87	0,000	0,062
NPTX2	Homo sapiens neuronal pentraxin II (NPTX2), mRNA [NM_002523]	866	7	-129,66	0,001	0,091
SERP2	Homo sapiens stress-associated endoplasmic reticulum protein family member 2 (SERP2), mRNA [NM_001010897]	1113	9	-118,81	0,000	0,055
NDN	Homo sapiens necdin homolog (mouse) (NDN), mRNA [NM_002487]	3225	28	-114,05	0,000	0,040
PCSK5	Homo sapiens proprotein convertase subtilisin/kexin type 5 (PCSK5), transcript variant 2, mRNA [NM_006200]	1249	13	-93,88	0,002	0,097
RADIL	Homo sapiens Ras association and DIL domains (RADIL), mRNA [NM_018059]	522	6	-88,12	0,000	0,040
EMX2OS	Homo sapiens EMX2 opposite strand/antisense RNA (non-protein coding) (EMX2OS), non-coding RNA [NR_002791]	498	6	-82,23	0,000	0,040
EMX2	Homo sapiens empty spiracles homeobox 2 (EMX2), transcript variant 1, mRNA [NM_004098]	492	6	-80,58	0,000	0,040
BACE2	Homo sapiens beta-site APP-cleaving enzyme 2 (BACE2), transcript variant a, mRNA [NM_012105]	6469	81	-79,8	0,000	0,046
SGCD	Homo sapiens sarcoglycan, delta (35kDa dystrophin-associated glycoprotein) (SGCD), transcript variant 1, mRNA [NM_000337]	990	14	-73,37	0,002	0,097
PLAC9	placenta-specific 9 [Source:HGNC Symbol;Acc:19255] [ENST00000372263]	618	9	-70,37	0,000	0,056
IRX2	Homo sapiens iroquois homeobox 2 (IRX2), transcript variant 1, mRNA [NM_033267]	613	9	-70,15	0,000	0,062
BACE2	Homo sapiens beta-site APP-cleaving enzyme 2 (BACE2), transcript variant a, mRNA [NM_012105]	6362	92	-69,5	0,000	0,040
CXCR7	Homo sapiens chemokine (C-X-C motif) receptor 7 (CXCR7), mRNA [NM_020311]	892	13	-69,19	0,001	0,092
ST6GAL2	Homo sapiens ST6 beta-galactosamide alpha-2,6-sialyltranferase 2 (ST6GAL2), transcript variant 1, mRNA [NM_032528]	363	6	-58,6	0,000	0,040
	Homo sapiens cDNA FLJ13585 fis, clone PLACE1009150. [AK023647]	434	8	-57,63	0,000	0,040
NKD2	Homo sapiens naked cuticle homolog 2 (Drosophila) (NKD2), mRNA [NM_033120]	5080	89	-56,91	0,001	0,084
SYNDIG1	Homo sapiens synapse differentiation inducing 1 (SYNDIG1), mRNA [NM_024893]	665	13	-53,13	0,000	0,055
ZSCAN18	Homo sapiens zinc finger and SCAN domain containing 18 (ZSCAN18), transcript variant 3, mRNA [NM_023926]	5746	109	-52,82	0,001	0,076
BEND5	Homo sapiens BEN domain containing 5 (BEND5), mRNA [NM_024603]	394	8	-51,35	0,000	0,040
F10	Homo sapiens coagulation factor X (F10), mRNA [NM_000504]	1502	30	-49,67	0,000	0,062
F13A1	Homo sapiens coagulation factor XIII, A1 polypeptide (F13A1), mRNA [NM_000129]	311	7	-48,13	0,000	0,040
ISLR	Homo sapiens immunoglobulin superfamily containing leucine-rich repeat (ISLR), transcript variant 1, mRNA [NM_005545]	10042	210	-47,91	0,000	0,040
KLHDC9	Homo sapiens kelch domain containing 9 (KLHDC9), transcript variant 2, mRNA [NM_001007255]	535	11	-47,27	0,001	0,078
SGCD	Homo sapiens sarcoglycan, delta (35kDa dystrophin-associated glycoprotein) (SGCD), transcript variant 2, mRNA [NM_172244]	634	14	-46,58	0,000	0,040
C18orf1	Homo sapiens chromosome 18 open reading frame 1 (C18orf1), transcript variant a2, mRNA [NM_181482]	312	7	-45,87	0,001	0,065

MEST	Homo sapiens mesoderm specific transcript homolog (mouse) (MEST), transcript variant 1, mRNA [NM_002402]	6120	146	-41,87	0,000	0,056
TTC12	Homo sapiens tetratricopeptide repeat domain 12 (TTC12), mRNA [NM_017868]	266	7	-40,58	0,000	0,056
CAPN6	Homo sapiens calpain 6 (CAPN6), mRNA [NM_014289]	231	6	-40,15	0,001	0,080
ELN	Homo sapiens elastin (ELN), transcript variant 1, mRNA [NM_000501]	443	11	-39,88	0,000	0,040
SLC38A4	Homo sapiens solute carrier family 38, member 4 (SLC38A4), transcript variant 1, mRNA [NM_018018]	233	6	-39,09	0,000	0,040
ECHDC3	Homo sapiens enoyl CoA hydratase domain containing 3 (ECHDC3), nuclear gene encoding mitochondrial protein, mRNA [NM_024693]	1995	51	-38,83	0,003	0,100
LOXL3	Homo sapiens lysyl oxidase-like 3 (LOXL3), mRNA [NM_032603]	7230	191	-37,92	0,000	0,062
MGAT3	Homo sapiens mannosyl (beta-1,4-)-glycoprotein beta-1,4-N-acetylglucosaminyltransferase (MGAT3), transcript variant 1, mRNA [NM_002409]	415	11	-37,44	0,002	0,094
SALL1	Homo sapiens sal-like 1 (Drosophila) (SALL1), transcript variant 1, mRNA [NM_002968]	207	6	-35,68	0,002	0,097
	argininosuccinate synthetase [human, Japanese classical citrullinemia patient A82, mRNA Partial Mutant, 91 nt]. [S73202]	1299	41	-31,81	0,000	0,065
		228	7	-31,37	0,000	0,040
AMOT	Homo sapiens angiomotin (AMOT), transcript variant 2, mRNA [NM_133265]	381	13	-29,76	0,000	0,056
ZNF423	Homo sapiens zinc finger protein 423 (ZNF423), mRNA [NM_015069]	219	7	-29,55	0,000	0,040
C8orf84	Homo sapiens chromosome 8 open reading frame 84 (C8orf84), mRNA [NM_153225]	1327	46	-29,1	0,002	0,095
TPD52	Homo sapiens tumor protein D52 (TPD52), transcript variant 1, mRNA [NM_001025252]	301	10	-29,09	0,001	0,072
THNSL2	Homo sapiens threonine synthase-like 2 (S. cerevisiae) (THNSL2), transcript variant 1, mRNA [NM_018271]	793	27	-29,03	0,002	0,097
SERPINF1	Homo sapiens serpin peptidase inhibitor, clade F (alpha-2 antiplasmin, pigment epithelium derived factor), member 1 (SERPINF1), mRNA [NM_002615]	2662	94	-28,42	0,000	0,062
GFRA1	Homo sapiens GDNF family receptor alpha 1 (GFRA1), transcript variant 2, mRNA [NM_145793]	2572	91	-28,36	0,000	0,056
EYA2	Homo sapiens eyes absent homolog 2 (Drosophila) (EYA2), transcript variant 1, mRNA [NM_005244]	1100	41	-26,69	0,000	0,040
PCSK5	proprotein convertase subtilisin/kexin type 5 [Source:HGNC Symbol;Acc:8747] [ENST00000376752]	300	11	-26,34	0,001	0,077
LOXL3	Homo sapiens lysyl oxidase-like 3 (LOXL3), mRNA [NM_032603]	986	39	-25,59	0,002	0,097
CDH15	Homo sapiens cadherin 15, type 1, M-cadherin (myotubule) (CDH15), mRNA [NM_004933]	275	11	-25,39	0,001	0,074
FBLN1	Homo sapiens fibulin 1 (FBLN1), transcript variant D, mRNA [NM_006486]	51993	2070	-25,12	0,001	0,079
SHISA2	Homo sapiens shisa homolog 2 (Xenopus laevis) (SHISA2), mRNA [NM_001007538]	511	21	-24,72	0,002	0,097
A2M	Homo sapiens alpha-2-macroglobulin (A2M), mRNA [NM_000014]	521	21	-24,69	0,000	0,062
QPCT	Homo sapiens glutaminyl-peptide cyclotransferase (QPCT), mRNA [NM_012413]	2703	110	-24,59	0,002	0,095
BNC1	Homo sapiens basonuclin 1 (BNC1), mRNA [NM_001717]	567	24	-23,64	0,000	0,046
C8orf84	Homo sapiens chromosome 8 open reading frame 84 (C8orf84), mRNA [NM_153225]	240	10	-23,45	0,001	0,075
DKFZP547L112	Homo sapiens mRNA; cDNA DKFZp547L112 (from clone DKFZp547L112). [AL512723]	207	9	-23,4	0,000	0,062
SLC12A7	Homo sapiens solute carrier family 12 (potassium/chloride transporters), member 7 (SLC12A7), mRNA [NM_006598]	10418	446	-23,38	0,000	0,040
		1490	65	-23,09	0,001	0,070
PTCHD1	Homo sapiens patched domain containing 1 (PTCHD1), mRNA [NM_173495]	274	13	-21,99	0,002	0,097
	Homo sapiens mRNA; cDNA DKFZp547I2016 (from clone DKFZp547I2016) [AL832534]	2559	120	-21,26	0,001	0,077
STMN3	Homo sapiens stathmin-like 3 (STMN3), mRNA [NM_015894]	22125	1066	-20,75	0,000	0,056

EYA2	Homo sapiens eyes absent homolog 2 (Drosophila) (EYA2), transcript variant 1, mRNA [NM_005244]	622	31	-20,41	0,000	0,040
COL15A1	Homo sapiens collagen, type XV, alpha 1 (COL15A1), mRNA [NM_001855]	335	17	-20,05	0,003	0,097
		721	36	-19,96	0,001	0,075
		262	13	-19,9	0,001	0,075
HPS1	Homo sapiens Hermansky-Pudlak syndrome 1 (HPS1), transcript variant 1, mRNA [NM_000195]	428	22	-19,34	0,000	0,060
ROR2	Homo sapiens receptor tyrosine kinase-like orphan receptor 2 (ROR2), mRNA [NM_004560]	893	51	-17,59	0,001	0,077
PBX4	Homo sapiens pre-B-cell leukemia homeobox 4 (PBX4), transcript variant 1, mRNA [NM_025245]	289	17	-17,24	0,001	0,078
EMILIN2	Homo sapiens elastin microfibril interfacer 2 (EMILIN2), mRNA [NM_032048]	12298	762	-16,14	0,001	0,077
ZNF135	Homo sapiens zinc finger protein 135 (ZNF135), transcript variant 2, mRNA [NM_003436]	219	14	-16,09	0,000	0,051
RTN1	Homo sapiens reticulon 1 (RTN1), transcript variant 1, mRNA [NM_021136]	394	25	-16,05	0,000	0,044
	Q4LUJ0_9BURK (Q4LUJ0) LrgB-like protein, partial (4%) [THC2783834]	646	41	-15,85	0,001	0,092
DNAH10	Homo sapiens dynein, axonemal, heavy chain 10 (DNAH10), mRNA [NM_207437]	224	14	-15,72	0,001	0,065
ROR1	Homo sapiens receptor tyrosine kinase-like orphan receptor 1 (ROR1), transcript variant 1, mRNA [NM_005012]	1843	124	-14,83	0,002	0,095
ZNF135	Homo sapiens zinc finger protein 135 (ZNF135), transcript variant 2, mRNA [NM_003436]	409	28	-14,74	0,002	0,096
SLC7A14	Homo sapiens solute carrier family 7 (orphan transporter), member 14 (SLC7A14), mRNA [NM_020949]	2361	161	-14,71	0,002	0,097
HHIPL1	Homo sapiens HHIP-like 1 (HHIPL1), transcript variant 1, mRNA [NM_001127258]	919	64	-14,39	0,000	0,040
GPR27	G protein-coupled receptor 27 [Source:HGNC Symbol;Acc:4482] [ENST00000304411]	252	18	-14,19	0,001	0,091
CARD9	Homo sapiens caspase recruitment domain family, member 9 (CARD9), transcript variant 1, mRNA [NM_052813]	426	30	-13,99	0,002	0,095
NUDT10	Homo sapiens nudix (nucleoside diphosphate linked moiety X)-type motif 10 (NUDT10), mRNA [NM_153183]	858	66	-13	0,000	0,046
IL17RD	Homo sapiens interleukin 17 receptor D (IL17RD), mRNA [NM_017563]	3351	300	-11,17	0,003	0,100
ACOX2	Homo sapiens acyl-CoA oxidase 2, branched chain (ACOX2), mRNA [NM_003500]	467	42	-11,11	0,000	0,056
GFRA1	Homo sapiens GDNF family receptor alpha 1 (GFRA1), transcript variant 2, mRNA [NM_145793]	376	34	-11,04	0,001	0,067
IGDCC4	Homo sapiens immunoglobulin superfamily, DCC subclass, member 4 (IGDCC4), mRNA [NM_020962]	1327	126	-10,5	0,003	0,100
SEMA3D	Homo sapiens sema domain, immunoglobulin domain (Ig), short basic domain, secreted, (semaphorin) 3D (SEMA3D), mRNA [NM_152754]	223	22	-10,14	0,000	0,062

88 gene symbols
