# Supplementary material for: Establishment of a Conditionally Immortalized Wilms Tumor Cell Line with a Homozygous WT1 Deletion within a Heterozygous 11p13 Deletion and UPD Limited to 11p15
Source: PLoS One. 2016 May 23;11(5):e0155561. doi: 10.1371/journal.pone.0155561 (PMC4876997; doi:10.1371/journal.pone.0155561)
Supplement: S3 Table — (PDF) [file pone.0155561.s018.pdf]

GeneSymbol	Description	Wilms10_mean	imWilms10_mean	imWilms10_vs_Wilms10_mean.fc	imWilms10_vs_Wilms10_p	Combined.FDR
FMO3	Homo sapiens flavin containing monooxygenase 3 (FMO3), transcript variant 2, mRNA [NM_001002294]	6	4270	712,45	0,000	0,048
MAGEC2	Homo sapiens melanoma antigen family C, 2 (MAGEC2), mRNA [NM_016249]	21	10285	490,55	0,000	0,050
PCLO	Homo sapiens piccolo (presynaptic cytomatrix protein) (PCLO), transcript variant 2, mRNA [NM_014510]	15	5357	370,51	0,001	0,070
CD70	Homo sapiens CD70 molecule (CD70), mRNA [NM_001252]	82	29049	355,85	0,000	0,062
EMR1	Homo sapiens egf-like module containing, mucin-like, hormone receptor-like 1 (EMR1), mRNA [NM_001974]	7	1592	244,92	0,000	0,046
FAM133A	Homo sapiens family with sequence similarity 133, member A (FAM133A), transcript variant 2, mRNA [NM_173698]	6	1276	218,74	0,000	0,040
PCLO	Homo sapiens piccolo (presynaptic cytomatrix protein) (PCLO), transcript variant 2, mRNA [NM_014510]	18	3366	191,52	0,000	0,040
DPYD	Homo sapiens dihydropyrimidine dehydrogenase (DPYD), transcript variant 1, mRNA [NM_000110]	10	1846	188,22	0,000	0,056
FMO3	Homo sapiens flavin containing monooxygenase 3 (FMO3), transcript variant 2, mRNA [NM_001002294]	7	1224	187,44	0,000	0,062
ALDH1A1	Homo sapiens aldehyde dehydrogenase 1 family, member A1 (ALDH1A1), mRNA [NM_000689]	15	2466	166,39	0,002	0,095
	DB728175 RIKEN full-length enriched human cDNA library, hypothalamus Homo sapiens cDNA clone H033001B22 3', mRNA sequence [DB728175]	7	995	145,19	0,000	0,050
BATF	Homo sapiens basic leucine zipper transcription factor, ATF-like (BATF), mRNA [NM_006399]	7	934	142,04	0,000	0,040
GRB14	Homo sapiens growth factor receptor-bound protein 14 (GRB14), mRNA [NM_004490]	18	1979	110,33	0,000	0,040
FAM133A	Homo sapiens family with sequence similarity 133, member A (FAM133A), transcript variant 2, mRNA [NM_173698]	13	1416	106,82	0,001	0,086
DPYD	Homo sapiens dihydropyrimidine dehydrogenase (DPYD), transcript variant 1, mRNA [NM_000110]	6	604	96,97	0,000	0,040
SLC38A11	Homo sapiens solute carrier family 38, member 11 (SLC38A11), transcript variant 2, mRNA [NM_173512]	7	527	80,28	0,000	0,046
PTGER3	Homo sapiens prostaglandin E receptor 3 (subtype EP3) (PTGER3), transcript variant 4, mRNA [NM_198714]	9	639	74,12	0,000	0,040
	Q5W0G2_HUMAN (Q5W0G2) Ankyrin repeat domain 18B, partial (11%) [THC2519054]	7	521	71,24	0,000	0,040
MUC15	Homo sapiens mucin 15, cell surface associated (MUC15), transcript variant 2, mRNA [NM_145650]	7	469	68,92	0,000	0,040
MRAP2	Homo sapiens melanocortin 2 receptor accessory protein 2 (MRAP2), mRNA [NM_138409]	13	860	68,2	0,001	0,070
SPANXA1	Homo sapiens sperm protein associated with the nucleus, X-linked, family member A1 (SPANXA1), mRNA [NM_013453]	802	51768	64,57	0,000	0,062
CXorf57	Homo sapiens chromosome X open reading frame 57 (CXorf57), transcript variant 1, mRNA [NM_018015]	28	1690	60,26	0,001	0,065
ZSCAN4	Homo sapiens zinc finger and SCAN domain containing 4 (ZSCAN4), mRNA [NM_152677]	6	329	60,25	0,000	0,040
CHRDL1	Homo sapiens chordin-like 1 (CHRDL1), transcript variant 1, mRNA [NM_001143981]	55	3292	59,49	0,001	0,092
RAVER1	Homo sapiens ribonucleoprotein, PTB-binding 1 (RAVER1), mRNA [NM_133452]	870	49810	57,27	0,000	0,040
		495	27849	56,31	0,000	0,056
LOC643201	Homo sapiens cDNA clone IMAGE:5171181. [BC034407]	7	357	54,54	0,000	0,040
UGT2B7	Homo sapiens UDP glucuronosyltransferase 2 family, polypeptide B7 (UGT2B7), mRNA [NM_001074]	7	360	53,88	0,001	0,091
ANKRD18B	Homo sapiens ankyrin repeat domain 18B (ANKRD18B), mRNA [NM_001244752]	6	327	53,12	0,000	0,040
SLC34A3	Homo sapiens solute carrier family 34 (sodium phosphate), member 3 (SLC34A3), transcript variant 3, mRNA [NM_080877]	421	22375	53,1	0,000	0,058
TNXB	Homo sapiens tenascin XB (TNXB), transcript variant XB, mRNA [NM_019105]	658	34274	52,06	0,000	0,048
SLFN11	Homo sapiens schlafen family member 11 (SLFN11), transcript variant 5, mRNA [NM_152270]	33	1682	51,4	0,000	0,049
COBLL1	Homo sapiens COBL-like 1 (COBLL1), mRNA [NM_014900]	27	1389	50,96	0,001	0,091
SPANXA1	Homo sapiens sperm protein associated with the nucleus, X-linked, family member A1 (SPANXA1), mRNA [NM_013453]	581	29491	50,75	0,000	0,040
CLGN	Homo sapiens calmegin (CLGN), transcript variant 1, mRNA [NM_004362]	58	2901	49,72	0,000	0,062
		45	2136	47,39	0,000	0,040
SFRP1	Homo sapiens secreted frizzled-related protein 1 (SFRP1), mRNA [NM_003012]	676	31654	46,85	0,001	0,067
SSX4B	Homo sapiens synovial sarcoma, X breakpoint 4B (SSX4B), transcript variant 1, mRNA [NM_001034832]	6	266	46,78	0,000	0,040

SPANXD	Homo sapiens SPANX family, member D (SPANXD), mRNA [NM_032417]	505	23277	46,11	0,000	0,040
C1orf229	Homo sapiens chromosome 1 open reading frame 229 (C1orf229), mRNA [NM_207401]	581	26532	45,64	0,000	0,062
DSEL	Homo sapiens dermatan sulfate epimerase-like (DSEL), mRNA [NM_032160]	11	484	42,95	0,000	0,062
		111	4713	42,39	0,000	0,040
VNN1	Homo sapiens vanin 1 (VNN1), mRNA [NM_004666]	7	275	42,34	0,001	0,065
DNER	Homo sapiens delta/notch-like EGF repeat containing (DNER), mRNA [NM_139072]	10	409	40,65	0,000	0,040
LOC92659	Homo sapiens uncharacterized LOC92659 (LOC92659), non-coding RNA [NR_015454]	770	31207	40,51	0,000	0,040
SCN4B	Homo sapiens sodium channel, voltage-gated, type IV, beta (SCN4B), transcript variant 1, mRNA [NM_174934]	73	2965	40,46	0,000	0,040
FCRLA	Homo sapiens Fc receptor-like A (FCRLA), transcript variant 2, mRNA [NM_032738]	30	1185	39,71	0,002	0,095
SFRP1	Homo sapiens secreted frizzled-related protein 1 (SFRP1), mRNA [NM_003012]	1880	72267	38,44	0,000	0,063
FOXB1	Homo sapiens forkhead box B1 (FOXB1), mRNA [NM_012182]	342	12897	37,67	0,000	0,040
	Homo sapiens, clone IMAGE:6155889, mRNA. [BC043411]	43	1598	37,35	0,002	0,097
GRIN1	Homo sapiens glutamate receptor, ionotropic, N-methyl D-aspartate 1 (GRIN1), transcript variant NR1-3, mRNA [NM_007327]	600	22181	36,99	0,001	0,072
GSTA2	Homo sapiens glutathione S-transferase alpha 2 (GSTA2), mRNA [NM_000846]	6	233	36,97	0,001	0,088
MFAP5	Homo sapiens microfibrillar associated protein 5 (MFAP5), mRNA [NM_003480]	803	29652	36,92	0,002	0,095
PAGE5	Homo sapiens P antigen family, member 5 (prostate associated) (PAGE5), transcript variant 1, mRNA [NM_130467]	7	241	36,28	0,000	0,062
OR52K3P	Homo sapiens clone IMAGE:110749 mRNA sequence. [AF143328]	12	444	35,85	0,000	0,060
SOX17	Homo sapiens SRY (sex determining region Y)-box 17 (SOX17), mRNA [NM_022454]	556	19871	35,72	0,000	0,040
SOX1	Homo sapiens SRY (sex determining region Y)-box 1 (SOX1), mRNA [NM_005986]	2042	72527	35,52	0,000	0,062
CDH10	Homo sapiens cadherin 10, type 2 (T2-cadherin) (CDH10), mRNA [NM_006727]	18	639	34,9	0,000	0,062
		26	862	33,77	0,000	0,056
PAGE2	Homo sapiens P antigen family, member 2 (prostate associated) (PAGE2), mRNA [NM_207339]	7	230	31,94	0,000	0,040
MRAP2	Homo sapiens melanocortin 2 receptor accessory protein 2 (MRAP2), mRNA [NM_138409]	7	207	31,69	0,000	0,040
	nq82c12.s1 NCI_CGAP_Co9 Homo sapiens cDNA clone IMAGE:1158838 3', mRNA sequence [AA639753]	123	3860	31,5	0,001	0,091
ZNF575	Homo sapiens zinc finger protein 575 (ZNF575), mRNA [NM_174945]	860	26528	30,84	0,000	0,060
	Homo sapiens cDNA FLJ38626 fis, clone HEART2009599. [AK095945]	1153	34692	30,09	0,000	0,051
CDH24	Homo sapiens cadherin 24, type 2 (CDH24), transcript variant 1, mRNA [NM_022478]	2308	68677	29,76	0,000	0,062
LRMP	Homo sapiens lymphoid-restricted membrane protein (LRMP), transcript variant 1, mRNA [NM_006152]	9	251	29,53	0,000	0,065
NCAM2	Homo sapiens neural cell adhesion molecule 2 (NCAM2), mRNA [NM_004540]	8	246	29,32	0,001	0,091
	Q412F2_KINRA (Q412F2) MOSC, partial (10%) [THC2481836]	287	8324	28,96	0,000	0,048
		1018	28957	28,45	0,000	0,062
TRIM43	Homo sapiens tripartite motif containing 43 (TRIM43), mRNA [NM_138800]	102	2857	28	0,000	0,060
ATP6V0A4	Homo sapiens ATPase, H+ transporting, lysosomal V0 subunit a4 (ATP6V0A4), transcript variant 1, mRNA [NM_020632]	40	1111	27,6	0,000	0,062
CNTNAP3B	Homo sapiens contactin associated protein-like 3B (CNTNAP3B), mRNA [NM_001201380]	66	1820	27,56	0,000	0,046
GPR150	Homo sapiens G protein-coupled receptor 150 (GPR150), mRNA [NM_199243]	3976	107772	27,1	0,000	0,056
ZSCAN4	Homo sapiens zinc finger and SCAN domain containing 4 (ZSCAN4), mRNA [NM_152677]	25	675	26,9	0,000	0,040
F11R	Homo sapiens F11 receptor (F11R), mRNA [NM_016946]	77	2028	26,39	0,002	0,097
		10	254	25,71	0,000	0,063
GABBR2	Homo sapiens gamma-aminobutyric acid (GABA) B receptor, 2 (GABBR2), mRNA [NM_005458]	35	908	25,7	0,000	0,040
		1229	30534	24,85	0,001	0,091
	Homo sapiens cDNA FLJ31085 fis, clone IMR321000037. [AK055647]	11	279	24,52	0,001	0,070
AGTR1	Homo sapiens angiotensin II receptor, type 1 (AGTR1), transcript variant 4, mRNA [NM_031850]	82	1898	23,13	0,002	0,097
		450	10290	22,87	0,001	0,070
TNFSF9	Homo sapiens tumor necrosis factor (ligand) superfamily, member 9 (TNFSF9), mRNA [NM_003811]	67	1504	22,57	0,001	0,080
MYRIP	Homo sapiens myosin VIIA and Rab interacting protein (MYRIP), mRNA [NM_015460]	19	428	22,27	0,000	0,040

LOC92249	Homo sapiens uncharacterized LOC92249 (LOC92249), non-coding RNA [NR_015353]	1584	34718	21,92	0,000	0,040
RGS16	Homo sapiens regulator of G-protein signaling 16 (RGS16), mRNA [NM_002928]	149	3264	21,89	0,001	0,066
MGC45922	Homo sapiens uncharacterized LOC284365 (MGC45922), non-coding RNA [NR_038359]	1094	23896	21,84	0,002	0,096
CYP24A1	Homo sapiens cytochrome P450, family 24, subfamily A, polypeptide 1 (CYP24A1), nuclear gene encoding mitochondrial protein, transcript variant 1, mRNA [NM_000782]	10	218	21,61	0,001	0,068
LINC00304	Homo sapiens long intergenic non-protein coding RNA 304 (LINC00304), non-coding RNA [NR_024347]	3287	70567	21,47	0,000	0,062
	RST1823 Athersys RAGE Library Homo sapiens cDNA, mRNA sequence [BG182941]	67	1426	21,29	0,000	0,062
VNN1	Homo sapiens vanin 1 (VNN1), mRNA [NM_004666]	16	346	21,16	0,000	0,045
JAM2	Homo sapiens junctional adhesion molecule 2 (JAM2), mRNA [NM_021219]	66	1393	21,01	0,000	0,065
CLCA2	Homo sapiens chloride channel accessory 2 (CLCA2), mRNA [NM_006536]	11	228	20,9	0,000	0,040
		515	10741	20,87	0,000	0,062
LOC388630	Homo sapiens UPF0632 protein A (LOC388630), mRNA [NM_001194986]	2484	51279	20,64	0,001	0,092
NEUROG3	Homo sapiens neurogenin 3 (NEUROG3), mRNA [NM_020999]	983	20218	20,57	0,002	0,096
LHX1	Homo sapiens LIM homeobox 1 (LHX1), mRNA [NM_005568]	184	3719	20,19	0,002	0,097
CYP26B1	Homo sapiens cytochrome P450, family 26, subfamily B, polypeptide 1 (CYP26B1), mRNA [NM_019885]	32	645	20,17	0,000	0,045
		1215	24288	19,99	0,001	0,080
ZFPM2	Homo sapiens zinc finger protein, multitype 2 (ZFPM2), mRNA [NM_012082]	38	758	19,88	0,002	0,097
TNFSF9	Homo sapiens tumor necrosis factor (ligand) superfamily, member 9 (TNFSF9), mRNA [NM_003811]	64	1215	19,09	0,000	0,062
		461	8707	18,89	0,001	0,070
KISS1R	Homo sapiens KISS1 receptor (KISS1R), mRNA [NM_032551]	1035	19494	18,83	0,001	0,075
NEUROG1	Homo sapiens neurogenin 1 (NEUROG1), mRNA [NM_006161]	464	8585	18,52	0,001	0,066
LOC145694	PREDICTED: Homo sapiens hypothetical LOC145694 (LOC145694), miscRNA [XR_109210]	173	3191	18,48	0,000	0,062
SCIN	Homo sapiens scinderin (SCIN), transcript variant 2, mRNA [NM_033128]	191	3483	18,28	0,001	0,091
SYN1	Homo sapiens synapsin I (SYN1), transcript variant Ib, mRNA [NM_133499]	2562	46812	18,27	0,001	0,075
CSTA	Homo sapiens cystatin A (stefin A) (CSTA), mRNA [NM_005213]	123	2193	17,84	0,001	0,091
BCAM	Homo sapiens basal cell adhesion molecule (Lutheran blood group) (BCAM), transcript variant 1, mRNA [NM_005581]	1022	18225	17,84	0,003	0,100
PARD6G	Homo sapiens par-6 partitioning defective 6 homolog gamma (C. elegans) (PARD6G), mRNA [NM_032510]	6544	116206	17,76	0,000	0,062
		1835	32180	17,54	0,000	0,062
NLRP11	Homo sapiens NLR family, pyrin domain containing 11 (NLRP11), mRNA [NM_145007]	13	226	17,23	0,000	0,055
		313	5275	16,84	0,000	0,053
CCNO	Homo sapiens cyclin O (CCNO), mRNA [NM_021147]	65	1086	16,71	0,000	0,062
FOXE1	H.sapiens HFKH4 mRNA for fork head like protein. [X94553]	231	3739	16,2	0,002	0,096
FBXL17	Homo sapiens F-box and leucine-rich repeat protein 17 (FBXL17), mRNA [NM_001163315]	6072	98142	16,16	0,001	0,078
LOC284344	Homo sapiens uncharacterized LOC284344 (LOC284344), non-coding RNA [NR_033888]	855	13609	15,92	0,002	0,094
RGS16	Homo sapiens regulator of G-protein signaling 16 (RGS16), mRNA [NM_002928]	44	693	15,84	0,000	0,062
NMU	Homo sapiens neuromedin U (NMU), mRNA [NM_006681]	42	661	15,76	0,000	0,049
		254	3967	15,62	0,002	0,097
GBP5	Homo sapiens guanylate binding protein 5 (GBP5), transcript variant 1, mRNA [NM_052942]	32	491	15,56	0,000	0,040
GJA3	Homo sapiens gap junction protein, alpha 3, 46kDa (GJA3), mRNA [NM_021954]	13	206	15,35	0,000	0,040
LOC440040	Homo sapiens glutamate receptor, metabotropic 5 pseudogene (LOC440040), non-coding RNA [NR_027044]	66	1001	15,24	0,000	0,062
		3720	56524	15,2	0,002	0,097
AGTR1	Homo sapiens mRNA for angiotensin II type 1b receptor, complete cds. [D13814]	87	1311	15,09	0,002	0,096
LOC572558	Homo sapiens uncharacterized LOC572558 (LOC572558), non-coding RNA [NR_015423]	27	405	14,79	0,000	0,062
EVX1	Homo sapiens even-skipped homeobox 1 (EVX1), mRNA [NM_001989]	430	6233	14,49	0,002	0,097
SNORA78	Homo sapiens, clone IMAGE:5221276, mRNA, partial cds. [BC028232]	3298	47194	14,31	0,002	0,097
ITGA7	Homo sapiens integrin, alpha 7 (ITGA7), transcript variant 2, mRNA [NM_002206]	771	10999	14,27	0,000	0,056
		11897	169088	14,21	0,000	0,051
		641	9114	14,21	0,002	0,097

	Q5VZL5_HUMAN (Q5VZL5) Zinc finger protein 262, complete [THC2723346]	1639	23170	14,14	0,000	0,052
		1932	27144	14,05	0,002	0,097
	Q34Z38_9GAMM (Q34Z38) Outer membrane efflux protein precursor, partial (5%) [THC2678411]	2010	28182	14,02	0,001	0,068
ATP10B	ATPase, class V, type 10B [Source:HGNC Symbol;Acc:13543] [ENST00000326831]	4154	57990	13,96	0,000	0,062
CYP4F2	Homo sapiens cytochrome P450, family 4, subfamily F, polypeptide 2 (CYP4F2), mRNA [NM_001082]	374	5179	13,85	0,002	0,097
MYO5C	Homo sapiens myosin VC (MYO5C), mRNA [NM_018728]	47	642	13,81	0,003	0,100
BAX	Homo sapiens BCL2-associated X protein (BAX), transcript variant delta, mRNA [NM_138763]	1168	16068	13,75	0,000	0,062
		5944	81607	13,73	0,001	0,077
JAM2	junctional adhesion molecule 2 [Source:HGNC Symbol;Acc:14686] [ENST00000480456]	32	434	13,73	0,001	0,092
FOXE1	Homo sapiens forkhead box E1 (thyroid transcription factor 2) (FOXE1), mRNA [NM_004473]	49	651	13,44	0,001	0,067
EN2	Homo sapiens engrailed homeobox 2 (EN2), mRNA [NM_001427]	3404	45517	13,37	0,001	0,091
AGAP3	Homo sapiens ArfGAP with GTPase domain, ankyrin repeat and PH domain 3 (AGAP3), transcript variant 2, mRNA [NM_001042535]	4593	60689	13,21	0,000	0,053
		5170	67901	13,13	0,001	0,065
SPATA2L	Homo sapiens spermatogenesis associated 2-like (SPATA2L), mRNA [NM_152339]	2379	31217	13,12	0,002	0,095
PSG2	Homo sapiens pregnancy specific beta-1-glycoprotein 2 (PSG2), mRNA [NM_031246]	818	10491	12,82	0,001	0,080
	Q2YU51_STAAB (Q2YU51) Glutamyl-tRNA amidotransferase subunit A , partial (5%) [THC2728011]	11404	145738	12,78	0,000	0,062
TRIM53P	Homo sapiens tripartite motif containing 53, pseudogene (TRIM53P), non-coding RNA [NR_028346]	40	498	12,6	0,002	0,094
		3771	47497	12,59	0,001	0,091
RTN3	Homo sapiens reticulon 3 (RTN3), transcript variant 4, mRNA [NM_201430]	10567	130994	12,4	0,000	0,062
NPBWR1	Homo sapiens neuropeptides B/W receptor 1 (NPBWR1), mRNA [NM_005285]	421	5204	12,36	0,000	0,055
LOC100507316	PREDICTED: Homo sapiens hypothetical LOC100507316, transcript variant 1 (LOC100507316), miscRNA [XR_133507]	348	4284	12,31	0,002	0,097
PSG7	Homo sapiens pregnancy specific beta-1-glycoprotein 7 (gene/pseudogene) (PSG7), transcript variant 1, mRNA [NM_002783]	213	2565	12,06	0,000	0,058
		433	5203	12	0,002	0,094
DSEL	Homo sapiens dermatan sulfate epimerase-like (DSEL), mRNA [NM_032160]	104	1241	11,96	0,002	0,094
CDKN2A	Homo sapiens cyclin-dependent kinase inhibitor 2A (melanoma, p16, inhibits CDK4) (CDKN2A), transcript variant 3, mRNA [NM_058197]	4899	58502	11,94	0,002	0,097
INPP5D	Homo sapiens inositol polyphosphate-5-phosphatase, 145kDa (INPP5D), transcript variant 1, mRNA [NM_001017915]	70	826	11,83	0,002	0,094
CNTNAP3	Homo sapiens contactin associated protein-like 3 (CNTNAP3), mRNA [NM_033655]	42	489	11,59	0,001	0,086
	Q3BCG3_9ARAC (Q3BCG3) Tubuliform spidroin (Fragment), partial (5%) [THC2721785]	43	498	11,55	0,001	0,078
NXPH3	Homo sapiens neurexophilin 3 (NXPH3), mRNA [NM_007225]	45	506	11,37	0,001	0,070
	Homo sapiens cDNA clone IMAGE:5301690. [BC039411]	152	1706	11,2	0,000	0,058
ATAD3C	Homo sapiens ATPase family, AAA domain containing 3C (ATAD3C), mRNA [NM_001039211]	2504	27871	11,13	0,002	0,097
	Q411X0_KINRA (Q411X0) Regulatory protein, LuxR:Response regulator receiver, partial (5%) [THC2538856]	20343	224351	11,03	0,000	0,058
PGM5	Homo sapiens phosphoglucomutase 5 (PGM5), mRNA [NM_021965]	40	442	11,02	0,000	0,062
	chromosome 6 open reading frame 220 [Source:HGNC Symbol;Acc:21553] [ENST00000369123]	411	4524	11,01	0,002	0,096
NKX2-8	Homo sapiens NK2 homeobox 8 (NKX2-8), mRNA [NM_014360]	1005	11022	10,96	0,002	0,097
LOC440461	Homo sapiens Rho GTPase activating protein 27 pseudogene (LOC440461), non-coding RNA [NR_027283]	4019	43811	10,9	0,000	0,062
KLF16	Homo sapiens Kruppel-like factor 16 (KLF16), mRNA [NM_031918]	11443	124766	10,9	0,001	0,069
LOC389607	PREDICTED: Homo sapiens hypothetical LOC389607 (LOC389607), miscRNA [XR_110088]	803	8734	10,88	0,000	0,046
CD36	CD36=collagen type I/thrombospondin receptor {one exon} [human, mRNA Partial, 369 nt]. [S67044]	42	450	10,83	0,001	0,072
GLTPD1	Homo sapiens glycolipid transfer protein domain containing 1 (GLTPD1), mRNA [NM_001029885]	973	10520	10,82	0,001	0,080
NRG1	Homo sapiens neuregulin 1 (NRG1), transcript variant GGF2, mRNA [NM_013962]	790	8537	10,81	0,001	0,075
FOXE1	Homo sapiens forkhead box E1 (thyroid transcription factor 2) (FOXE1), mRNA [NM_004473]	50	539	10,74	0,001	0,083
PSG6	Homo sapiens pregnancy specific beta-1-glycoprotein 6 (PSG6), transcript variant 1, mRNA [NM_002782]	2749	29067	10,57	0,002	0,094
PCDH7	Homo sapiens protocadherin 7 (PCDH7), transcript variant a, mRNA [NM_002589]	430	4524	10,53	0,000	0,046
		11924	124764	10,46	0,001	0,072
DLGAP3	Homo sapiens discs, large (Drosophila) homolog-associated protein 3 (DLGAP3), mRNA [NM_001080418]	8211	85521	10,42	0,002	0,097
PSG11	Homo sapiens pregnancy specific beta-1-glycoprotein 11 (PSG11), transcript variant 1, mRNA [NM_002785]	870	8923	10,26	0,001	0,091
	SPAST_HUMAN (Q9UBP0) Spastin, partial (3%) [THC2657348]	4252	43012	10,11	0,001	0,086
		1865	18844	10,11	0,002	0,097
FRMD4A	Homo sapiens FERM domain containing 4A (FRMD4A), mRNA [NM_018027]	384	3859	10,06	0,000	0,058
ZFPM1	Homo sapiens zinc finger protein, multitype 1 (ZFPM1), mRNA [NM_153813]	9923	99747	10,05	0,002	0,097
	Homo sapiens cDNA FLJ34952 fis, clone NTONG2000531. [AK092271]	4026	40301	10,01	0,002	0,094

182 gene symbols
