# Supplementary material for: Establishment of a Conditionally Immortalized Wilms Tumor Cell Line with a Homozygous WT1 Deletion within a Heterozygous 11p13 Deletion and UPD Limited to 11p15
Source: PLoS One. 2016 May 23;11(5):e0155561. doi: 10.1371/journal.pone.0155561 (PMC4876997; doi:10.1371/journal.pone.0155561)
Supplement: S3 Fig — (PDF) [file pone.0155561.s003.pdf]

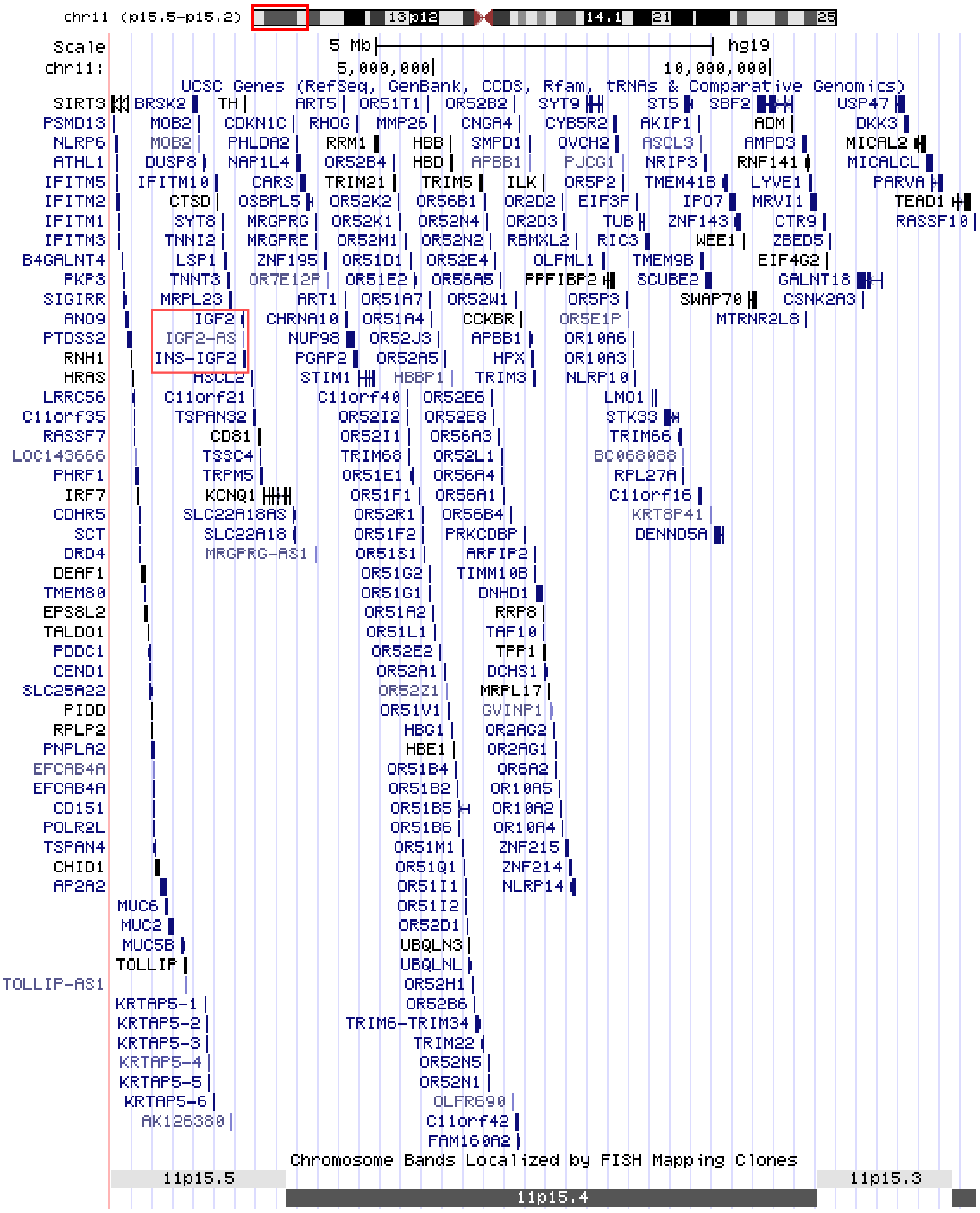

Figure S3. The size and position of the UDP in 11p15

The terminal UPD region of 11p15pter is shown with the genes mapping to this segment. The position of the *IGF2* gene labeled with a red box
